# Unravelling the Spatial and Temporal Plasticity of Eelgrass Meadows

**DOI:** 10.3389/fpls.2021.664523

**Published:** 2021-05-20

**Authors:** Chiara M. Bertelli, James C. Bull, Leanne C. Cullen-Unsworth, Richard K. F. Unsworth

**Affiliations:** ^1^Department of Biosciences, Swansea University, Swansea, United Kingdom; ^2^Sustainable Places Research Institute, Cardiff University, Cardiff, United Kingdom

**Keywords:** *Zostera marina*, plasticity, bioindicator, water quality, nutrient, resilience

## Abstract

The phenotypic plasticity of seagrasses enables them to adapt to changes in environmental conditions and withstand or recover from disturbance. This plasticity was demonstrated in the large variation recorded throughout a suite of bioindicators measured within *Zostera marina* meadows around Wales and SW England, United Kingdom. Short-term spatial data were analysed alongside long-term monitoring data to determine which bioindicators best described the status of eelgrass meadows subjected to a range of environmental and anthropogenic drivers. Shoot density, leaf length, leaf nutrients (C:N ratio, %N, %P) including stable isotope of δ^13^C and δ^15^N provided insight into the longer-term status of the meadows studied and a good indication of the causes of long-term decline. Meadows ranged from those in the Isles of Scilly with little evidence of impact to those in Littlewick in Milford Haven, Wales that showed the highest levels of impacts of all sites. Bioindicators at Littlewick showed clear warning signs of nutrient loading reflected in the long-term decline in shoot density, and prevalence of wasting disease. This study highlights the need for continuous consistent monitoring and the benefits of using extra tools in the form of shoot nutrient analysis to determine causes of decline.

## Introduction

Seagrass is protected under International, European, and United Kingdom legislation and monitoring of meadows has been integrated into management and Water Framework Directives as an indicator of good ecological status of coastal waters (Krause-jensen et al., 2005; Foden and Brazier, 2007; Marbà et al., 2013; de los Santos et al., 2019). This has led to an increase in monitoring of seagrass meadows around Europe in recent decades (de los Santos et al., 2019). However, the diverse range of seagrass indicators used (Marbà et al., 2013) and the difference in frequency of monitoring surveys make it difficult to make assumptions on the true status of these vital habitats. Baselines for monitoring have major implications for how the interpretation of the status of seagrass meadows is or has altered over time. Monitoring enables the management and protection of seagrass meadows from direct existing or potential impacts, such as reductions in water quality. This ultimately improves the overall health and resilience of the seagrass to increasing threats from climate change (Björk et al., 2008). As an important carbon store in the marine environment, it is even more pertinent that seagrass meadows are protected and, where viable, restored so that they can continue to absorb CO_2_ from the atmosphere (Röhr et al., 2018).

*Zostera marina* meadows around the British Isles are degraded in status, with estimations of 25–49% decline in the last 35 years (Hiscock et al., 2005; Jackson et al., 2013) although recent evidence has this value at 92%, a loss of approximately 75,000 ha (Green et al., 2021). To be able to set criteria for monitoring and mitigation strategies within management plans, it is important to understand environmental drivers of seagrass meadows. Environmental conditions such as light, temperature and depth will affect many physiological, morphological, and structural parameters of seagrass meadows (Martínez-Crego et al., 2008). The plasticity of seagrasses enables them to adapt to changes in environmental conditions and in turn to withstand certain levels of disturbances (Short and Wyllie-Echeverria, 1996). These changes can be used as bioindicators of reduced light levels, nutrient input and other impacts that can be attributed to anthropogenic disturbance or other causes for decline in water quality. Detailed studies of seagrass responses to light reduction have revealed a number of consistent and robust bioindicators such as reductions in shoot density, biomass, growth and production, and shorter narrower leaves (McMahon et al., 2013). Above ground biomass is reduced in this way in order to reduce the respiratory and energetic costs that come from the production and maintenance of new leaves (Fourqurean and Zieman, 1991; Collier et al., 2012). Chlorophyll content of leaves can increase under low light, with the chlorophyll *a:b* ratio lowering to increase photosynthetic efficiency (Silva et al., 2013). However, if light stress is prolonged, the production of more chloroplasts may prove too costly and resulting in the rapid decline in photosynthetic performance within a relatively short time-frame (Ralph and Gademann, 2005; Bité et al., 2007). Based on such evidence it can be assumed that the morphology and physiology of *Z. marina* can provide an insight into the overall light environments *in situ* and hence the status of coastal waters (Dennison et al., 1993).

Leaf biochemistry of seagrass can also be used to signify changes in ecological health of coastal waters from eutrophication (Fourqurean et al., 1997; Jones and Unsworth, 2016). Such studies in the United Kingdom found most seagrass to be in a poor condition, with nutrient values in excess of global averages (Jones and Unsworth, 2016). Additionally, shoot C:N ratio and the stable isotope of carbon, δ^13^C have both been identified as a robust and early indicator of light stress (McMahon et al., 2013), with C:N shown to have a positive relationship with seagrass cover (McKenzie et al., 2011). Also, the stable isotope of nitrogen δ^15^N in seagrass can be used to identify anthropogenic sources of nutrient inputs from agricultural or urban effluents (Lepoint et al., 2004; Jones et al., 2018), providing indications of the source of eutrophication threat to the ecosystem (Short et al., 1995; Lee et al., 2004).

In order to understand the status of seagrass, monitoring of abiotic factors such as temperature, turbidity and light are also important (Jackson et al., 2013; Burton et al., 2015; McDonald et al., 2016) as natural environmental processes also effect seagrass growth. Temperature has been found to effect the morphology of *Z. marina* with wider leaved plants being found in areas where the annual temperature fluctuation is small such as the Scilly Isles (Den Hartog, 1970). Also, *Z. marina* growing in higher wave exposure will have significant morphological differences to plants growing where relative wave exposure is lower (Krause-Jensen et al., 2003). Changes in depth limits of seagrass growth is one of the bioindicators used to inform the WFD of changes to water quality as deeper maximum depth limits suggest clearer waters (Dennison and Alberte, 1985; Dennison, 1987; Krause-jensen et al., 2005). Density will also be lower at increased depths as a response to lower light in order to reduce self-shading and reduce respiratory demand (Collier et al., 2007). This supports the need for monitoring a number of robust bioindicators alongside abiotic parameters within seagrass meadows when assessing status. When bioindicators at the meadow or plant-scale change, hypothesising the potential drivers is compromised by gaps in explanatory environmental and seagrass data. Specifically, it is important to determine if changes are natural processes such as yearly fluctuations in sunlight hours and sea surface temperature, or are being caused by anthropogenic sources such as light limitation caused by nutrient loading (Rasheed and Unsworth, 2011). The need for detailed reference conditions need to be taken into account for such changes to be properly assessed (Krause-jensen et al., 2005).

The aims of this study were to investigate the plasticity of *Z. marina* using a suite of morphological and physiological indicators over a range of environmental conditions and hypothesise that these responses can be used to explain changes occurring in these meadows over time using available long-term monitoring survey data. These sites include one in the Isles of Scilly used as a low impact “reference site” thought to have reduced anthropogenic pressures.

## Materials and Methods

### Seagrass Condition

**FIGURE 1:**
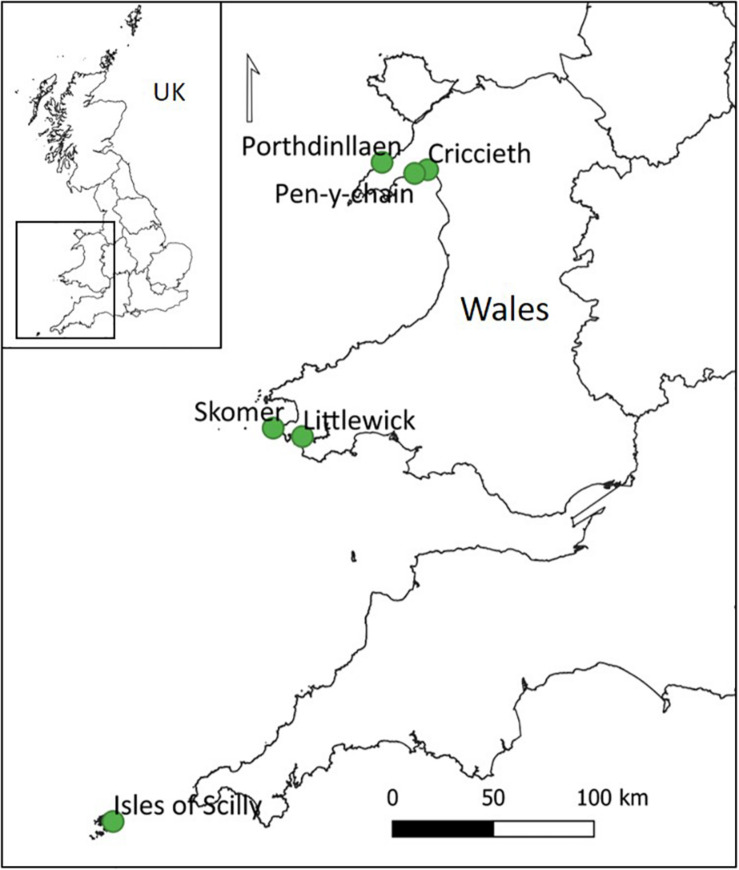
Locations of seagrass sites surveyed around Wales and on the Isles of Scilly, United Kingdom.

Six *Z. marina* meadows around the coast of Wales and the Isles of Scilly (United Kingdom) were assessed for morphological and physiological factors. The sites were as follows: Littlewick bay 51.706°N, 5.067°W (Milford Haven), North Haven 51.738°N, 5.280°W (Skomer), Pen-y-chain 52.899°N, 4.322°W, Criccieth 52.917°N, 4.227°W and Porthdinllaen 52.943°N, 4.565°W (Llyn Peninsula) and Little Arthur 49.948°N, 6.265°W within the Isles of Scilly (Figure 1). All sites were surveyed in August and September 2016.

At each site a PAR logger (Odyssey, Dataflow systems Ltd) and a temperature logger (Tinytag aquatic 2) were deployed and left *in situ* for a month to record light availability and temperature in the middle of the seagrass meadows. The light logger was placed vertically attached on the mooring block at 50 cm above the seabed so it would be recording at the top of the canopy, and to avoid shading. A Secchi disk was used to measure turbidity, and depth was recorded using a dive computer (Suunto zoop) on the survey days and corrected to Chart Datum using tidal prediction software (POLTIPS v3, Bell, 2016). Wave energy index for each site was calculated using data taken from EMODnet seabed habitats portal which provides data on variables that influence habitat type taken from various survey sources. For each site the three grid squares (0.3 km resolution) closest to the survey position that contained wave energy data were averaged to give an overall value.

At each Welsh site, the mid-meadow and meadow edges were identified from previous site data collection and drop-down camera work (Nagle, 2013; Brown, 2015; Burton et al., 2015). Ten 50 cm × 50 cm quadrat were placed haphazardly through the middle of the meadow, perpendicular to the shore. Within each quadrat, 25 cm × 25 cm area of seagrass was removed, with shoots being cut just at the level of the substrate and cut shoots placed in separate zip lock bags. Where visibility was good enough, a Go-Pro^®^Hero 4 camera attached to the top of the quadrat frame was used to video the quadrats. This allowed extra data to be collected including percentage coverage of seagrass and algae which were analysed from video footage. This was repeated at the edge of the meadow in order to get a good representation overall. At Pen-y-chain and Criccieth, the seagrass was found to be relatively patchy and a distinct edge was not found owing to poor visibility, so only measurements through the middle of the meadow were possible.

All shoots collected were counted and each leaf measured. Shoot measurements included leaf length (taken from top of sheath to tip of leaf), leaf width, epiphyte and wasting disease cover. Leaf length was measured with a measuring tape to the nearest mm, and leaf width was measured using callipers to the nearest 0.1 mm. Canopy height was estimated by taking the maximum leaf length of each shoot. Epiphyte and wasting disease cover was scored between 0 and 5 for each leaf (whereby “0” = 0%, 0% < “1” ≤ 2%, 2% < “2” ≤ 25%, 25% < “3” ≤ 50%, 50% < “4” ≤ 75%, and 75% < “5” ≤ 100%) based on the index developed for wasting disease (Burdick et al., 1993).

Shoot data for the Isles of Scilly site, Little Arthur, was obtained from Natural England annual surveys which follow a comparable method outlined in Lobelle et al. (2013) and Potouroglou et al. (2014). This allowed for the inclusion of metric data from the 2016 annual survey to be included into this study.

#### Leaf Nutrient Analysis

Samples of seagrass were taken from each of the sites and leaves were separated, scraped free of epiphytes, and dried. The dried seagrass was ground up with a pestle and mortar to a fine homogenous powder. Samples were sent to OEA laboratories Limited for analysis of the % composition of Carbon, Nitrogen and Phosphorus by weight using a continuous flow isotope ratio mass spectrometer (Sercon 20–20 IRMS coupled to Thermo EA1110 elemental analyser). The ratios of stable isotopes 13C to 12C (δ^13^C) and 15N to 14N (δ^15^N) were also determined to give values which can indicate light availability, nutrient availability and anthropogenic sources of nutrients (Jennings et al., 1997; Lepoint et al., 2004). Leaf nutrient data for the Isles of Scilly was obtained from a previous study by Jones et al. (2018).

### Long-Term Data Analysis

Four long-term monitoring datasets for Skomer (Burton et al., 2019), Littlewick (Hiscock, 1987; Irving and Worley, 2000; Nagle, 2013; Unsworth et al., 2017a), Porthdinllaen (Project Seagrass, 2019), and Isles of Scilly (Lobelle et al., 2013; Alotaibi et al., 2019) were collated and standardised. All comparable data were extracted for analysis for temporal changes and trends.

### Statistical Analysis

All averages are reported ± Standard Deviation. GLM is a flexible method of analysis that can be used on different types of data including count data (shoot density) and continuous data (leaf lengths) without being limited by the assumptions of normally distributed data (Crawley, 2005). For leaf lengths and widths, GLMs with Gamma errors were used which is most appropriate for continuous data such as measurements (Crawley, 2005; Zuur et al., 2009). For epiphyte, wasting disease, seagrass cover and algae cover, GLM with binomial errors which is appropriate for proportion data. All scores and percentages were converted to proportions (0–1). For over or underdispersed data whereby the residual deviance was higher or lower than the degree of freedom, quasi-binomial GLM was used instead to correct for this, making the models more conservative with lower chance of type 1 error (Crawley, 2005). For count data, shoot density and number of leaves, Poisson (or quasi-poisson for overdispersion) GLM with log link was used which ensures all fitted values are positive (Crawley, 2005). All GLM were carried out using R Studio (R version 4.0.2). Model comparisons were made using a likelihood ratios test with and without site as a factor to assess significance of site on the parameter. Where appropriate, Tukey pairwise comparisons between sites were undertaken using the “glht” function in the “multcomp” package in R studio. This analysis was also carried on long-term datasets using year as a factor.

Principal Components Analysis (PCA) was carried out using shoot level data for maximum leaf length, leaf width, epiphytes and wasting disease. All data were scaled before analysis. As not all data were collected at the same resolution separate PCA were conducted including shoot metric data, quadrat level data (to include shoot density), and meadow-scale data (to compare nutrient data). PCA conducted on quadrat level data to include shoot density and leaves per shoot. Leaf nutrients and stable isotopes (C:N, %N, %P, δ^15^N, δ^13^C) were analysed using PCA separately alongside average shoot density to see if they were having an effect on shoot count as has been found in other studies. Owing to cost of nutrient analysis, sample number for nutrients was limited therefore a separate PCA was conducted to visualise similarities between meadows. Principal components with eigenvalues > 1.0 were considered, and eigenfactors or variable coefficients ≤ –0.3, or ≥ 0.3 were selected. All PCA was carried out using Primer-e (version 6).

## Results

### Seagrass Condition

**FIGURE 2:**
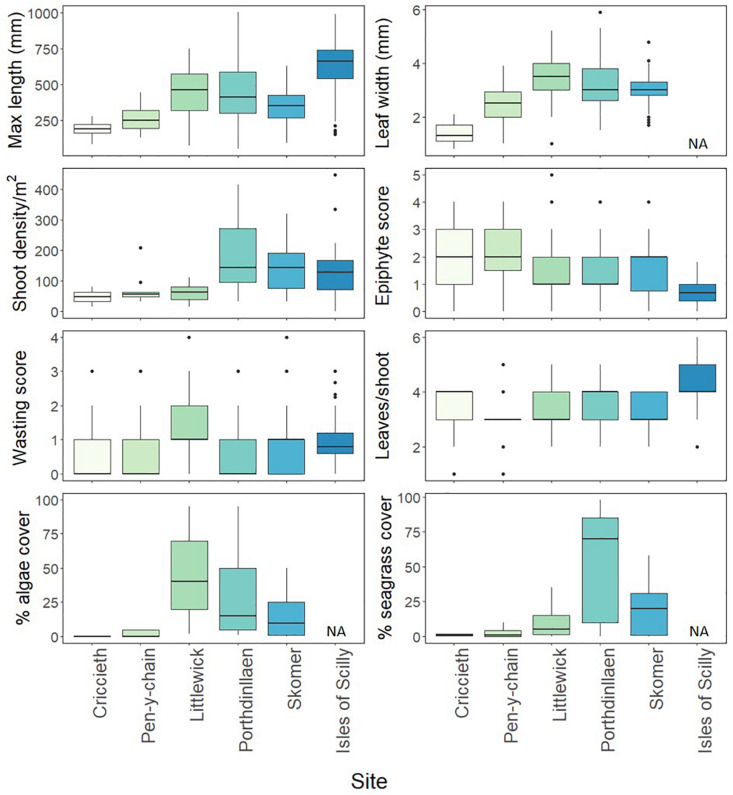
Seagrass shoot and meadow characteristics measured at different seagrass sites. The box-whisker represents the median (line) and interquartile range (box) with additional 1.5 × interquartile range shown as whisker. Outliers are shown as points outside the box-whisker plots. Algae and seagrass cover taken from drop-down camera footage of quadrats taken at each site except Isles of Scilly (*n* ≥ 40 per meadow except Criccieth where *n* = 12 due to poor visibility).

The morphological plasticity of seagrass throughout our six survey sites from 2016 was highly variable and likelihood ratios tests showed that site as a factor had a significant effect on all metrics (Supplementary Table A.1). Leaf length was significantly longer in the Isles of Scilly (630.68 ± 162.71 mm) than any other site (Figure 2 and Supplementary Table A.2). Littlewick had the widest leaves (3.41 ± 0.78 mm) although width data was not available for Isles of Scilly. Density was highest in Porthdinllaen (189.18 ± 109.43 shoots per m^2^) along with Skomer and Isles of Scilly, all of which were found to have significantly higher shoot densities than other sites (Figure 2 and Supplementary Table A.2). Criccieth and Pen-y-chain were found to have similar shoot densities to Littlewick, albeit with shorter and narrower leaves (Figure 2 and Supplementary Table A.2). Wasting disease was significantly higher in Littlewick (1.29 ± 0.51) than Porthdinllaen, Skomer, and Isles of Scilly (Figure 2 and Supplementary Table A.2) with the lowest scores in Porthdinllaen (0.47 ± 0.47). Pen-y-chain had the highest epiphyte score (2.12 ± 0.59) and the lowest scores were in the Isles of Scilly (0.67 ± 0.39, Figure 2 and Supplementary Table A.2) although most sites were not different from each other. Numbers of leaves per shoot were highest on the Isles of Scilly (4.38 ± 0.86) and significantly higher than all sites except for Porthdinllaen (Figure 2 and Supplementary Table A.2).

Seagrass cover and algae percentage cover from the drop-down camera varied significantly between the sites surveyed (no data for Isles of Scilly). Model comparisons found that site as a factor was found to having a significant effect on seagrass and algae cover (Supplementary Table A.1). Seagrass cover was significantly higher in Porthdinllaen (54.2 ± 37.69%) than all other sites (Figure 2 and Supplementary Tables A.1, A.2). Algae cover was highest in Littlewick (44.8 ± 28.51%, Figure 2 and Supplementary Table A.2), significantly higher cover than Skomer and Pen-y-chain (Figure 2 and Supplementary Table A.2).

#### Nutrient Analysis

Seagrass nutrient results show high levels of variability between sites (Table 1). Isles of Scilly had the lowest %P and δ^15^N content showing little, if any, evidence of nutrient enrichment from anthropogenic sources at this site. These nutrient parameters were found to be highest in seagrass from Littlewick indicating nutrient enrichment. Skomer, however, had the lowest C:N, δ^13^C and the highest %N suggesting light limitation and nutrient enrichment.

**TABLE 1:** Results from the elemental analysis of *Z. marina* leaf tissue taken from the study sites.

Site	%N	%P	C:N	δ^15^N	δ^13^C
Criccieth	2.23 ± 0.23	0.24 ± 0.03	15.87 ± 0.46	6.37 ± 0.33	-14.71 ± 0.22
Littlewick	2.27 ± 0.24	0.40 ± 0.04	18.98 ± 0.18	10.17 ± 0.1	-14.36 ± 0.31
Pen-y-chain	2.26 ± 0.13	0.29 ± 0.03	19.41 ± 0.82	7.60 ± 0.63	-13.69 ± 0.57
Porthdinllaen	2.22 ± 0.38	0.33 ± 0.04	21.09 ± 0.59	7.72 ± 0.05	-13.65 ± 0.59
Skomer	3.04 ± 0.19	0.33 ± 0.02	14.71 ± 0.18	8.03 ± 0.1	-16.90 ± 0.28
Isles of Scilly	2.76 ± 0.29	0.14 ± 0.01	20.56 ± 2.55	4.47 ± 0.97	n/a
*Study average*	*2.46* ± *0.36*	*0.29* ± *0.09*	*18.44* ± *2.44*	*6.71* ± *3.06*	-*14.66* ± *1.28*

*The stable isotope values for δ^15^N indicate the deviation of the isotopic composition relative to air. The isotope values for δ^13^C indicate the deviation of the isotopic composition relative to the Vienna PeeDee Belemnite (VPDB) standard. All values are unitless.*

#### Principal Components Analysis

Principal components analysis (PCA) was carried out on all available parameters measured at both shoot and quadrat levels for all Welsh sites. All of the shoot level metrics (width, length, epiphyte, and wasting score) were shown by PCA to be strongly contributing to the variability between the seagrass meadows (Supplementary Figure A.1A and Supplementary Table A.4) with all factors found to be significant across the first two components explaining over 80% of the variation. PC1 and shows a significant correlation between leaf length, width and wasting disease with all eigen factors over 0.3 (Supplementary Table A.4). The second PCA (Supplementary Figure A.1B) shows leaf length and width contribute strongly to explaining the variation between meadows in Wales at the quadrat level with PC1 and PC2 explaining nearly 65% of the variation. PC2 shows a strong positive correlation with epiphyte and wasting disease cover and a negative correlation with leaves per shoot. The third PCA was used to compare shoot nutrient data for each of the sites in Wales and plotted with shoot density and shows a higher level of clustering of sites. PC1 shows a strong positive correlation of C:N and isotope δ^13^C with decreasing %N (Supplementary Figure A.2C) demonstrating higher light availability (↑C:N, δ^13^C) with decreasing nutrient inputs (↓%N). PC2 (33.2% variation) shows a positive correlation with δ^15^N and %P, both of which would increase in seagrass meadows with nutrient loading. PC3 (18.6% variation) shows a positive correlation with δ^15^N but negative correlation with shoot density (Supplementary Table A.4) suggesting an increase in anthropogenic sourced nutrients having a negative effect on shoot density.

**FIGURE 3:**
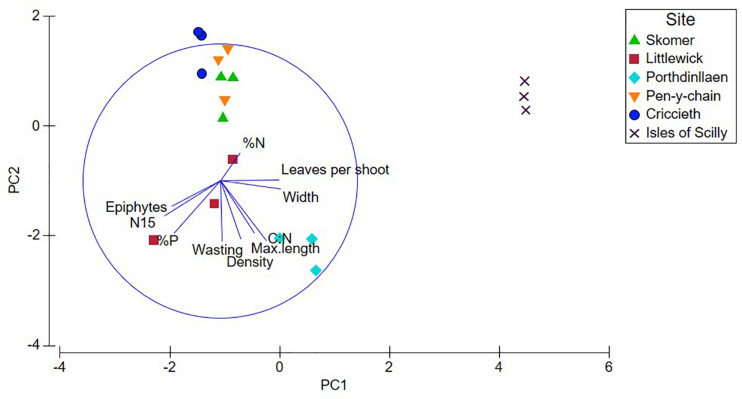
Principal Components Analysis (PCA) plots for shoot, nutrient, and stable isotope data for each site, plotted with shoot density and metrics (max. leaf length, width, and leaves per shoot). Nutrient data for Isles of Scilly provided from Jones et al. (2018).

Nutrient data available for the Isles of Scilly included all nutrient parameters (except for δ^13^C) and relevant shoot metrics and was therefore included in a fourth PCA (Figure 3 and Table 2). Epiphytes, δ^15^N and %P showed significant negative correlation with leaf length, width and leaves per shoot in PC1 (47% variation). Clustering of sites shown in Figure 3 shows the Isles of Scilly sharing no overlap with other sites particularly on PC1 axis, whereas Skomer, Pen-y-chain, and Criccieth show more similarity.

**TABLE 2:** Results from the Principal Components Analysis (PCA) carried out using available data from Welsh sites and Isles of Scilly for nutrient data, shoot metrics, and density.

PCA1—Shoot data	PC1	PC2	PC3
*Summary values*			
Eigenvalues	**4.72**	**2.05**	**1.33**
Percent variation	47.2	20.5	13.3
Cumulative percent variation	47.2	67.7	80.9
*Seagrass variables*			
Max. leaf length	**0.333**	**–0.437**	0.074
Leaf width	**0.440**	**–0.058**	0.075
Epiphyte	**–0.355**	**–0.186**	0.145
Wasting	0.013	**–0.440**	0.163
Leaves per shoot	**0.428**	0.008	0.062
%N	0.145	0.204	**0.750**
%P	**–0.338**	**–0.381**	0.176
C:N	0.245	**–0.383**	**–0.493**
δ^15^N	**–0.411**	–0.254	0.023
Density	0.147	**–0.424**	**0.317**

*Bold values show significant levels of eigenvalues (above 1 for principal components, and eigenfactors or variable coefficients ≤ –0.3, or ≥ 0.3).*

### Environmental Variables

Environmental variables are shown in Table 3. No data was available for the Isles of Scilly site. Pen-y-chain and Porthdinllaen were found to have the highest light availability based on PAR logger data, whereas light Criccieth had the lowest (Table 3). Temperature results showed little difference between sites so is likely having limited effect on the meadows that can be discerned from this short-term data (Table 3) Wave energy data shows the higher wave exposure effecting the seagrass at Criccieth and Pen-y-chain when compared to average results for Skomer, Porthdinllaen, and Littlewick. Criccieth and Pen-y-chain were also found to be considerable shallower than other sites with higher turbidity.

**TABLE 3:** Abiotic and environmental data collected for each site collected in August-September 2016, averages ± standard deviation.

Site	Light (PAR)	Temp (C°)	Wave energy (N.m^2^.s^–1^)	Turbidity—Secchi (m)	Max. depth (m)
Criccieth	391.42 ± 506.28	17.64 ± 0.31	160.45 ± 28.15	0.5 ± 0.01	2.5 ± 0.25
Littlewick	n/a	n/a	83.54 ± 46.49	1.65 ± 0.01	4 ± 0.45
Pen-y-chain	796.74 ± 875.16	17.76 ± 0.34	165.68 ± 39.1	1 ± 0.02	2.5 ± 0.32
Porthdinllaen	779.84 ± 702.83	16.53 ± 0.25	19.18 ± 9.1	5 ± 0.02	5.2 ± 0.39
Skomer	420.49 ± 324.84	16.07 ± 0.34	24.20 ± 3.2	6 ± 0.03	8.2 ± 0.46
Study average	595.89 ± 656.68	16.99 ± 0.78	90.61 ± 70.45	2.83 ± 2.23	4.48 ± 2.12

*Light data for each site is a daily average of PAR logged every 10 min. Temperature was also logged every 10 min. Depths were adjusted to Chart Datum. Wave energy was averaged from data taken from EMODnet (https://www.emodnet-seabedhabitats.eu/access-data/launch-map-viewer/).*

### Long-Term Changes

#### Shoot Density

**FIGURE 4:**
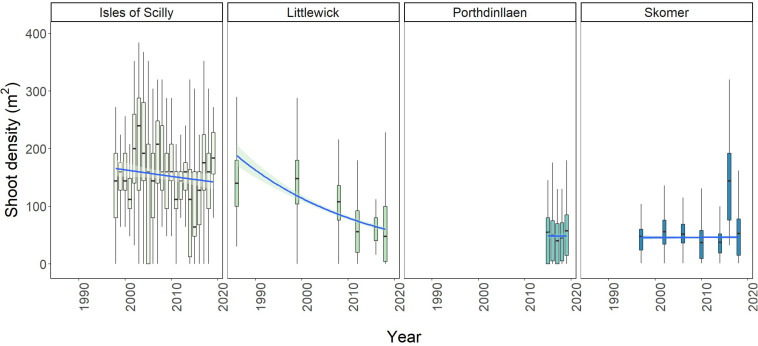
Boxplots showing change in average shoot density per m^2^ over time for Littlewick, Skomer, Porthdinllaen, and Isles of Scilly. The box-whisker represents the median (line) and interquartile range (box) with additional 1.5 × interquartile range shown as whiskers. Outliers not shown for clarity (data provided by NRW, Project Seagrass and Natural England respectively, with data from this study included for Skomer and Littlewick).

**FIGURE 5:**
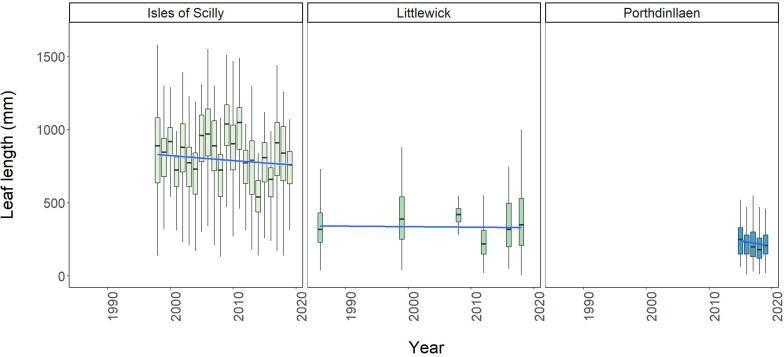
Boxplots showing change in average leaf length over time for Littlewick, Porthdinllaen, and average maximum leaf length for Isles of Scilly. The box-whisker represents the median (line) and interquartile range (box) with additional 1.5 × interquartile range shown as whisker and a temporal trendline in blue (GLM smooth with Gamma family), grey area shows 95% confidence. Outliers not shown for clarity (data provided by NRW, Project Seagrass, and Natural England respectively, with data from this study included for Littlewick).

Significant changes in shoot density with year were found at all sites except for Porthdinllaen (Supplementary Table A.54). For Littlewick, shoot density was found to be the highest in 1999 (141.39 ± 61.9, Figure 4). Shoot density has consistently decreased since surveys began (Figure 4) with the lowest density recorded in 2012 (Figure 4 and Supplementary Table A.6). Pairwise comparisons show that all years measured have significantly lower shoot density than 1986 and 1999. Most recent surveys (2012, 2016, and 2018) are also significantly lower than in 2008 (Supplementary Table A.5). For Skomer, seagrass densities show a different pattern with densities significantly increasing between 1997 and 2006 (Figure 4 and Supplementary Table A.6). The surveys in 2014 show the lowest overall density recorded (36.15 ± 22.04). Density was found to be highest in the 2016 survey (Figure 4 and Supplementary Table A.6), although there is less variability between monitored years (Figure 4). Seagrass shoot density in Porthdinllaen has shown little variation through time, with year having no effect on density for the years measured (*F* = 0.9984), *p* = 0.41, *df* = 580, 584). For the annual Isles of Scilly surveys, year was found to be having a significant effect on density (*F* = 3.791, *p* < 0.001, *df* = 495, 516). The highest average shoot counts overall were recorded in 2003 (256.64 ± 199.76 shoots m^2^) and the lowest shoot density was in 2015 (106.24 ± 93.17 shoots m^2^). The pairwise comparison showed that only the years 2002, 2003, and 2004 (with the highest densities recorded) were significantly higher than other years, with only 14 out of 231 pairwise comparisons showing significance (Supplementary Table A.5). Most years did not show significant differences, and shoot density appears to be relatively stable over time (Figure 5 and Supplementary Table A.5). The lowest shoot densities for Isles of Scilly were found to correlate with historic sunshine hour data taken the closest weather station data^1^ (Supplementary Figure A.2). The continuous annual monitoring in the Isles of Scilly allowed us to undertake this analyse there but not at other sites.

#### Leaf Length

Leaf length data was the only other comparable metric monitored long-term, and only available for Littlewick and Porthdinllaen in Wales, and the Isles of Scilly whereby maximum leaf lengths are measured (Figure 5). Model comparison demonstrated that leaf length at all three sites showed significant changes with year (Supplementary Table A.5). Leaf length in Littlewick has changed significantly over time, with the biggest overall increase in lengths recorded in 1999 followed by the largest decline in 2012 (Figure 5 and Supplementary Table A.6). The survey in 2016 did not record a significant change in leaf length, but 2018 data shows a significant increase (Supplementary Table A.6), back to similar lengths recorded in 1999 (Figure 5). For Porthdinllaen, since 2015 there is some decline in leaf length, with the biggest decline in 2018 (Figure 5 and Supplementary Table A.6), but lengths have increased somewhat by 2019 with pairwise comparisons showing a significant increase in length from 2015 to 2018 (Supplementary Table A.6). The seagrass in the Scilly Isles is showing significant fluctuations in leaf length, with the longest since monitoring started (in 1996) being in 2009 (994.16 ± 265.43 mm), and the shortest in 2014 (534.63 ± 155.47mm). Over time, leaf length appears relatively stable (Figure 5), however the results of the pairwise comparison showed significant differences between most years (156 out of 231 pairwise comparisons, Supplementary Table A.6).

#### Leaf Condition

**FIGURE 6:**
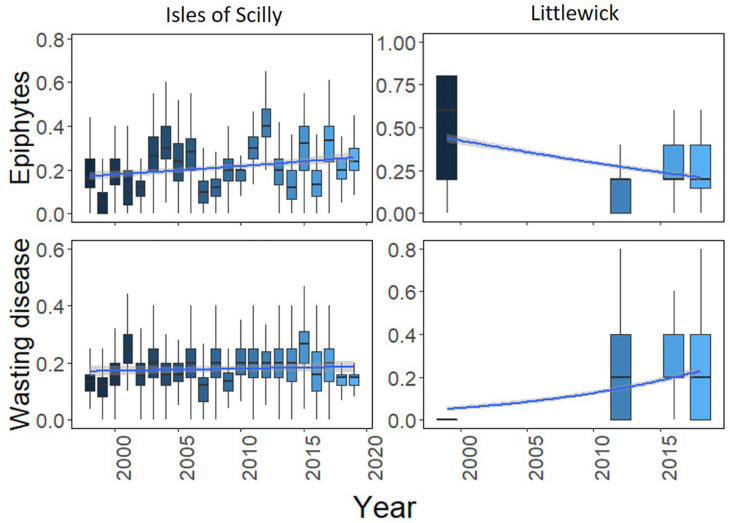
Boxplots showing change in leaf condition (epiphyte cover and wasting disease) over time for Littlewick and the Isles of Scilly. The box-whisker represents the median (line) and interquartile range (box) with additional 1.5 × interquartile range shown as whisker. Scale is as a proportion based on the original scores, with temporal trendline in blue (GLM smooth with binomial errors for proportion data) with 95% confidence in grey either side. Outliers have been taken out for clarity (data provided by NRW, and Natural England, with data from this study included for Littlewick for 2016).

Long-term shoot condition data was only available for Littlewick and the Isles of Scilly. For Littlewick, both epiphyte and wasting disease showed significant temporal changes, with a decrease in epiphytes and an increase in wasting disease cover over each year (Figure 6). Changes in epiphyte cover between years for the Isles of Scilly site fluctuate but with a slight increase over time. Wasting disease shows little variation with the only significant increases shown between a few years (Figure 6).

## Discussion

Here we provide a unique analysis of bioindicators of seagrass at spatial (short-term) and temporal (long-term) scales. The spatial study allowed for the measurement of a wide range of seagrass characteristics which can provide evidence of environmental drivers affecting the variation in seagrass plasticity and condition between different locations. The long-term study involving the analysis of data from monitored seagrass sites provides insight into the relative stability or instability of the meadows studied.

The plasticity of seagrasses enables them to adapt to changes in environmental conditions and to a degree withstand or recover from some level of anthropogenic disturbance (Short and Wyllie-Echeverria, 1996; Maxwell et al., 2014). At sites in Wales and SW England environmental and anthropogenic factors were found to influence this plasticity as demonstrated in the large variation found across a suite of seagrass of indicators.

All the bioindicators measured were found to describe significant amounts of variation between sites. The morphological and physiological bioindicators enabled differentiation in Wales between sites, with the extensive meadow at Porthdinllaen appearing to be the healthiest reflected by shoot morphology, condition and leaf biochemistry. This meadow was found to have the highest shoot density and cover, with leaf nutrient bioindicators indicating a higher light environment and lower nutrient loading. The long-term data and earlier studies validate this finding with the seagrass community found to be stable between years (Edwards et al., 2003; Morris et al., 2009). Although the temporal range of data for Porthdinllaen is limited, evidence exists that this site remains a stable eelgrass bed showing similar shoot density to the Isles of Scilly site.

Relatively high wave energy and turbidity were recorded as the principle drivers of the two shallowest meadows at Criccieth and Pen-y-chain. These meadows had the shortest and narrowest leaves and lowest shoot densities, a possible response to increased wave motion and risk of uprooting. Average temperatures measured over the survey period were over 1°C higher in these two shallow meadows than the other sites surveyed which is likely to have an effect on the respiratory demand of the plants. Higher variability in temperature in shallower waters will be contributing to the dynamism of the localised environment. Eelgrass from Criccieth was found to be in the poorest condition due to low shoot C:N, δ^13^C, shoot density and high epiphyte cover. The PAR levels measured were found to be lowest in Criccieth presumably due to increased turbidity via the resuspension of sediments from high wave energy. However, shoot nutrient analysis indicates low nutrient input at this site suggesting natural processes are having the biggest impact on seagrass condition. Pen-y-chain was found to have the highest PAR levels most likely due to shallow depth and lower turbidity, reflected by high shoot C:N and δ^13^C. Criccieth has been previously recorded as a sparse meadow (Edwards et al., 2003), suggesting it is somewhat dynamic owing to its physical environment.

Our bioindicator approach found low light differentiated the meadow at Skomer from other localities (low PAR, C:N, δ^13^C) even though superficially shoot density was similar to Porthdinllaen and the Isles of Scilly. This prognosis is verified by the long-term instability in the system. Low light maybe a natural phenomenon driven by elevated nitrogen due to run-off from the colonies of breeding seabirds that nest on the surrounding cliffs from April to June (Wilkie et al., 2001). This regular seasonal input of nutrients appears to be causing periodic reductions in the local light environment, causing seagrass here to be relatively dense but with shorter and narrower leaves. The long-term data shows this meadow to be fluctuating significantly but there is no steady decrease which suggests these changes could be attributed to natural fluctuations in yearly sunshine hours and short-term, seasonal light limitation from plankton blooms and epiphyte growth caused by nutrient run-off from seabird colonies.

By comparison, the bioindicators measured show the meadow at Littlewick is showing strong signs of anthropogenic impact. The shelter from wave action suggests the area should be conducive to seagrass growth, yet shoot densities are comparable to sites where wave action is much higher. The leaf condition and nutrient biondicators suggest that nutrient loading is impacting this meadow (highest δ^15^N, %P and wasting score) despite leaf length and width being high. This meadow was also found to have the highest percentage cover of algae. Other studies looking at the effects of eutrophication in eelgrass beds have also found increases in leaf length and a reduction in shoot density as a response to increased shading from opportunistic algae (Moore et al., 1996; Short and Burdick, 1996; Schmidt et al., 2012). High inorganic nitrogen (N_i_) in the water column can cause seagrasses to be more susceptible to infections from wasting disease as anti-microbial compounds are produced less to compensate for the synthesis of excess nitrogen in plant tissues (Short and Burdick, 1996; Burkholder et al., 2007). These factors combined strongly to imply that the seagrass meadow in Littlewick is under threat from eutrophic conditions and is undergoing a system shift from a seagrass dominated to macroalgae-dominated community. Long-term data for Littlewick supports this assumption, whereby leaf length has shown significant increases in most years, but shoot density is showing a steady significant decline. Wasting disease has also increased significantly since monitoring started.

Seagrass in Wales relative to the Isles of Scilly (IoS) as a reference site seagrass with limited anthropogenic impacts. Shoot densities and leaf widths in IoS are somewhat comparable with Skomer and Porthdinllaen, but the addition of shoot nutrient parameters (in this case C:N, δ^15^N, and %P) results in huge dissimilarities between meadows. Leaf length is significantly longer in Isles of Scilly which has been previously recognised as the longest eelgrass found in United Kingdom waters (Den Hartog, 1970; Jones and Unsworth, 2016). The increased water clarity of this archipelago is caused by the granite substrate and sediments that settle rapidly (Jackson et al., 2011) and the lack of large scale agriculture and urbanisation. This allows *Z. marina* to grow at greater depths with longer leaf lengths than other locations where turbidity reduces the maximum depth limit of seagrass growth (Nielsen et al., 2002). The lower impacts from terrestrial run-off are shown in the high C:N and lower %P and δ^15^N. The long-term yearly monitoring of the eelgrass meadows in the Isles of Scilly allows for fine-scale temporal changes to be shown. The main threats to seagrass around these remote islands is physical damage caused by boat moorings, anchoring and storms (Jackson et al., 2013; Bull and Kenyon, 2015; Unsworth et al., 2017b), not necessarily water quality issues. The data used for this study comes from the site that was found to be the least impacted and provided a good control site for comparison of status. The yearly monitoring of the Isles of Scilly allows for better evidence-based projections of long-term trends and changes, with shoot density showing much more stability than canopy height over time. It is likely that fluctuations are caused by changes in sunshine hours or other natural processes, with sunshine hours showing a positive correlation with shoot density for the Isles of Scilly (Supplementary Figure A.2). The slower response of shoot density to environmental stresses than other metrics raises the alarm for systems that are seeing continuous declines.

Density of the seagrass *Zostera marina* overall is showing some decline over the last two decades, providing evidence that seagrass in the United Kingdom is still somewhat degraded in state with no measurable upward trend of recovery as seen in some species such as *Z. noltii* (Bernard et al., 2007; Bertelli et al., 2018). The lowest densities appear to have been recorded between 2012 and 2015 which could be a United Kingdom wide response to natural processes such as significant changes in average recorded sunshine hours.

We also present strong evidence of significant and consistent long-term decline of one of Wales’ largest seagrass meadows at Littlewick in the Milford Haven Waterway. The increase in leaf length together with the reduction in density strongly indicate that Littlewick Bay is suffering from frequent and/or prolonged nutrient loading, to the point that natural environmental processes, such as fluctuations in sunshine hours, could be hidden. Milford Haven Waterway, which encompasses Littlewick, has been designated as being of moderate status and hypernutrified in terms of the WFD standards for nutrients (NRW, 2016). This is reflected in the high tissue nutrients found from the spatial study which explains this trend. By contrast, other sites have shown some increase in shoot density in the most recent years and an overall level of stability in density as seen in the Isles of Scilly, Porthdinllaen, and Skomer. Due to complexities of the factors influencing the resilience of seagrass meadows it is difficult to determine how close such a meadow is to a catastrophic tipping point, however, considerable long-term seagrass monitoring evidence globally indicates that once such a point is reached complete degradation and loss can be rapid (Waycott et al., 2009).

Shoot density is affected by numerous disturbances, including light limitation, nutrient loading, physical damage, temperature, or natural storm events, and therefore is one of the most important parameters that can be implemented into monitoring programmes. Consistent monitoring methods between sites can enable the identification of naturally occurring temporal trends that could be affecting structural responses or where trends are not consistent, indicate localised anthropogenic disturbances. Significant changes to shoot density should then justify the use of other robust bioindicators of stress to determine the causes of decline.

## Conclusion

This study demonstrates the high levels of plasticity exhibited by eelgrass to environmental conditions and the need for regular, consistent long-term monitoring of seagrass sites for significant declines to be detected. Structural bioindicators or responses such as shoot density, cover, biomass and extent are often included (one or all) in general seagrass monitoring programmes but do not integrate the use of bioindicators.

Our evidence indicates that where significant changes are detected such biochemical indicators can become powerful metrics for determining sources of declines. For sites where there is a lack of monitoring data, a suite of bioindicators and abiotic factors can be measured to interpret environmental conditions and provide meaningful understanding as to the status of those seagrasses that are potentially indicative of long-term trends. Left unchecked seagrass meadows are highly susceptible to degradation and loss, principally due to the development of a phase shift from seagrass to an algal dominated state. Our study provides a warning that such shifts may be likely at some, particularly as their resilience to future stressors is compromised by poor water quality. In conclusion we find that long-term monitoring of seagrasses is critical for helping inform management of such meadows to prevent catastrophic changes from occurring.

## Data Availability Statement

Publicly available datasets were analysed in this study. This data can be found here: Natural Resources Wales, https://naturalresources.wales/evidence-and-data/accessing-our-data/access-our-data-maps-and-reports/?lang=en and Natural England https://naturalengland-defra.opendata.arcgis.com/pages/accessing-data-services. Contains Natural Resources Wales information © Natural Resources Wales and database right. All rights reserved.

## Author Contributions

CB: contributed to the conceptualization, data curation, formal analysis, investigation, methodology, project administration, software, visualization, and writing—original draft preparation. LC-U and JB: contributed to the data resources and curation. RU: contributed to the conceptualization, supervision, validation, and writing—review and editing. All authors contributed to the article and approved the submitted version.

## Conflict of Interest

The authors declare that the research was conducted in the absence of any commercial or financial relationships that could be construed as a potential conflict of interest.

## Footnotes

1 Metoffice.gov.uk
