# Supplementary material for: Unravelling the Spatial and Temporal Plasticity of Eelgrass Meadows
Source: Front Plant Sci. 2021 May 20;12:664523. doi: 10.3389/fpls.2021.664523 (PMC8174302; doi:10.3389/fpls.2021.664523)
Supplement: Supplementary file 1 [file Data_Sheet_1.docx]

## Appendix

### **Figures**


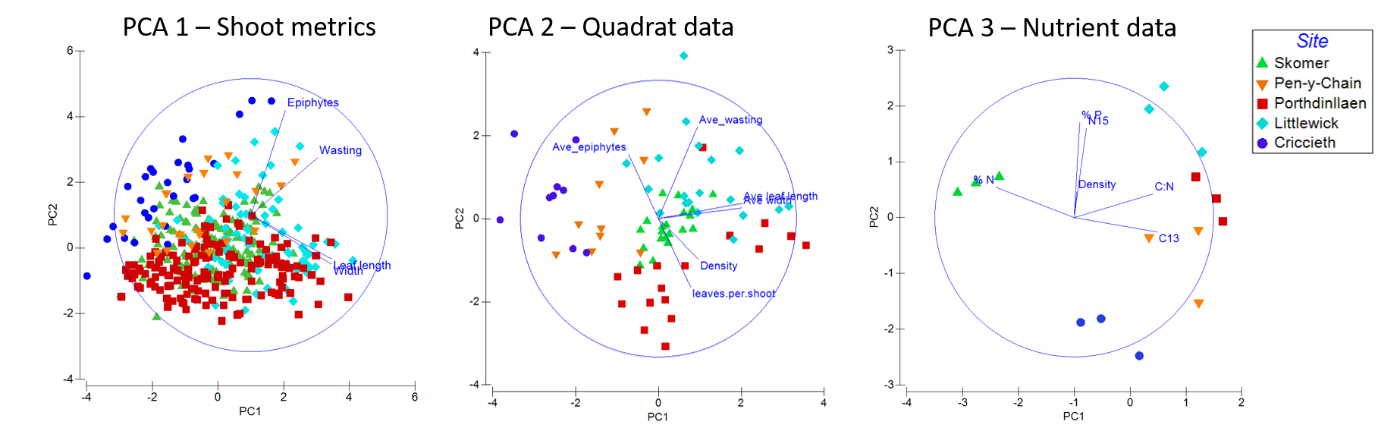


Figure A.1. Graphs of the three Principal Component Analysis plots carried out on data from each of the sites around Wales (1) shoot level metrics, (2) quadrat level metrics including shoot density and leaves per shoot, and (3) shoot nutrient and stable isotope, plotted with shoot density.


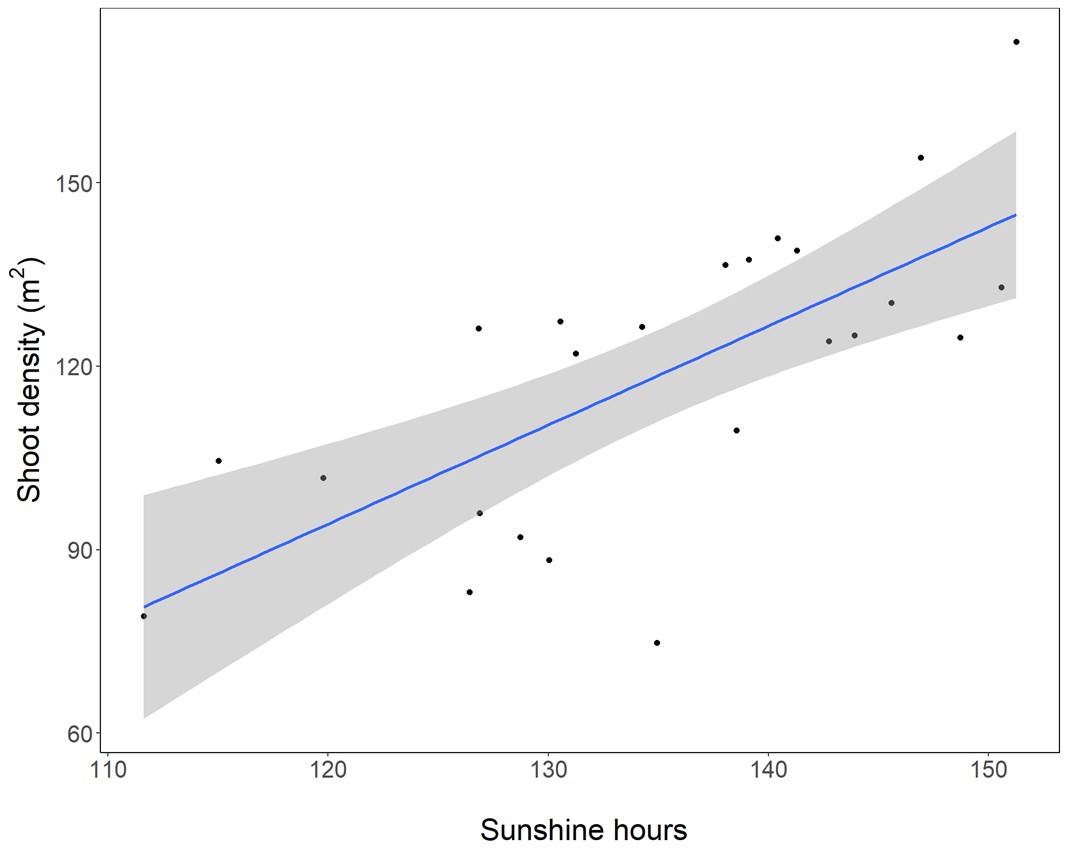


Figure A.2. Graph showing linear regression of yearly average shoot density (m^2^) from Isles of Scilly plotted against average sunshine hours per month for each year density data was available, taken from Met Office data recorded at nearby Cambourne weather station, Cornwall. The blue line shows linear trendline (linear model) with 95% confidence limits shaded in grey. Results from the linear regression shows a positive correlation (R2=0.505, F=22.4, p=<0.001).

### **Tables**

Table A.1. Analysis of Deviance table showing results of the likelihood ratios test for comparing GLM models with and without ‘Site’ to assess significance of test.

| Shoot density | | | | | | |
| --- | --- | --- | --- | --- | --- | --- |
| Model 1: density ~ 1 | | | | | | |
| Model 2: density ~ site | | | | | | |
|  | Resid. Df | Resid. Dev | Df | Deviance | F | Pr(>F) |
| 1 | 98 | 6322.6 |  |  |  |  |
| 2 | 93 | 4158.5 | 5 | 2164.1 | 9.6228 | **1.993e-07 ***** |
| No. of leaves | | | | | | |
| Model 1: no_lvs ~ 1 | | | | | | |
| Model 2: no_lvs ~ site | | | | | | |
|  | Resid. Df | Resid. Dev | Df | Deviance | Pr(>Chi) |  |
| 1 | 678 | 145.04 |  |  |  |  |
| 2 | 673 | 104.38 | 5 | 40.66 | **1.099e-07 ***** | |
| Leaf width | | | | | | |
| Model 1:leaf_width ~ site | | | | | | |
| Model 2: leaf_width ~ 1 | | | | | | |
|  | Resid. Df | Resid. Dev | Df | Deviance | F | Pr(>F) |
| 1 | 1670 | 92.069 |  |  |  |  |
| 2 | 1674 | 148.729 | -4 | -56.659 | 273.06 | **< 2.2e-16 ***** |
| Max. leaf length | | | | | | |
| Model 1: max_length ~ 1 | | | | | | |
| Model 2: max_length ~ site | | | | | | |
|  | Resid. Df | Resid. Dev | Df | Deviance | F | Pr(>F) |
| 1 | 682 | 156.22 |  |  |  |  |
| 2 | 677 | 91.637 | 5 | 64.582 | 111.93 | **< 2.2e-16 ***** |
| Wasting | | | | | | |
| Model 1: wasting_prop ~ 1 | | | | | | |
| Model 2: wasting_prop ~ site | | | | | | |
|  | Resid. Df | Resid. Dev | Df | Deviance | Pr(>Chi) |  |
| 1 | 1746 | 179.14 |  |  |  |  |
| 2 | 1741 | 146.06 | 5 | 33.082 | **3.626e-06 ***** | |
| Epiphytes | | | | | | |
| Model 1: epiphyte_prop ~ 1 | | | | | | |
| Model 2: epiphyte_prop ~ site | | | | | | |
|  | Resid. Df | Resid. Dev | Df | Deviance | F | Pr(>F) |
| 1 | 1861 | 496.29 |  |  |  |  |
| 2 | 1856 | 477.21 | 5 | 19.078 | 14.395 | **7.421e-14 ***** |
| Seagrass cover | | | | | | |
| Model 1: seagrass_cover ~ 1 | | | | | | |
| Model 2: seagrass_cover ~ site | | | | | | |
|  | Resid. Df | Resid. Dev | Df | Deviance | F | Pr(>F) |
| 1 | 235 | 125.944 |  |  |  |  |
| 2 | 231 | 68.337 | 4 | 57.607 | 50.371 | **<2.2e-16 ***** |
| Algae cover | | | | | | |
| Model 1: algae_cover ~ 1 | | | | | | |
| Model 2: algae_cover ~ site | | | | | | |
|  | Resid. Df | Resid. Dev | Df | Deviance | F | Pr(>F) |
| 1 | 235 | 129.78 |  |  |  |  |
| 2 | 231 | 108.65 | 4 | 21.132 | 11.264 | **2.291e-08 ***** |

Table A.2. Results from Generalized Linear Models (GLM) for shoot metrics (leaf length, width, epiphyte and wasting disease scores) and meadow data (shoot density and number of leaves per shoot). Gamma GLM was used for continuous measures (length and width), binomial GLM for proportion data (epiphyte and wasting scores, seagrass and algae % cover as proportion 0-1), and poisson or quasipoisson GLM for count data (shoot density and number of leaves).

| **Coefficients - Metric** | **Estimate** | **Std. Error** | **t-value** | **Pr(>\|t\|)** |
| --- | --- | --- | --- | --- |
| **Max. leaf length** formula=glm(max_leaf_length~site, family = Gamma (link=”log”)) | | | | |
| (Intercept) Criccieth | 5.25119 | 0.06308 | 83.243 | < 2e-16 *** |
| Littlewick | 0.85980 | 0.07428 | 11.575 | < 2e-16 *** |
| Pen-y-chain | 0.30616 | 0.08125 | 3.768 | 0.000179 *** |
| Porthdinllaen | 0.85557 | 0.06846 | 12.497 | < 2e-16 *** |
| Skomer | 0.59197 | 0.06834 | 8.662 | < 2e-16 *** |
| Isles of Scilly (Little Arthur) | 1.19561 | 0.06740 | 17.740 | < 2e-16 *** |
| **Leaf width** formula=glm(leaf_width~site, family = Gamma (link=”log”)) | | | | |
| (Intercept) Criccieth | 0.31316 | 0.02362 | 13.26 | <2e-16 *** |
| Littlewick | 0.93534 | 0.02749 | 34.02 | <2e-16 *** |
| Pen-y-chain | 0.59841 | 0.03065 | 19.53 | <2e-16 *** |
| Porthdinllaen | 0.82705 | 0.02531 | 32.68 | <2e-16 *** |
| Skomer | 0.78844 | 0.02551 | 30.90 | <2e-16 *** |
| **Epiphytes** formula=glm(epiphyte~site, family = binomial) | | | **z-value** |  |
| (Intercept) Criccieth | -1.0479 | 0.2365 | -4.431 | 9.39e-06 *** |
| Littlewick | -0.8245 | 0.3133 | -2.632 | 0.008501 ** |
| Pen-y-chain | -0.6641 | 0.2922 | -2.272 | 0.023062 * |
| Porthdinllaen | -0.2471 | 0.316 | -0.782 | 0.434137 |
| Skomer | -1.0085 | 0.2682 | -3.761 | 0.000169 *** |
| Isles of Scilly (Little Arthur) | -0.6495 | 0.2646 | -2.455 | 0.014092 * |
| **Wasting disease** formula=glm(wasting~site, family = binomial) | | |  |  |
| (Intercept) Criccieth | -3.449 | 0.597 | -5.777 | 7.59e-09 *** |
| Littlewick | -1.2557 | 0.9513 | -1.32 | 0.1869 |
| Pen-y-chain | 1.1674 | 0.6339 | 1.842 | 0.0655 . |
| Porthdinllaen | -0.511 | 0.9089 | -0.562 | 0.574 |
| Skomer | -0.6738 | 0.6911 | -0.975 | 0.3296 |
| Isles of Scilly (Little Arthur) | -0.2895 | 0.6614 | -0.438 | 0.6615 |
| **Shoot density** formula=glm(shoot_density~site, family = quasipoisson) | | | **t-value** |  |
| (Intercept) Criccieth | 3.8373 | 0.3113 | 12.325 | < 2e-16 *** |
| Littlewick | 0.3216 | 0.3660 | 0.879 | 0.38180 |
| Pen-y-Chain | 0.4169 | 0.4010 | 1.040 | 0.30124 |
| Porthdinllaen | 1.4054 | 0.3330 | 4.220 | 5.68e-05 *** |
| Skomer | 1.1100 | 0.3360 | 3.304 | 0.00136 ** |
| Isles of Scilly (Little Arthur) | 1.1228 | 0.3326 | 3.375 | 0.00108 ** |
| **Leaves per shoot** formula=glm(leaves_per_shoot~site, family = poisson) | | | **z-value** |  |
| (Intercept) Criccieth | 1.20039 | 0.10370 | 11.576 | <2e-16 *** |
| Littlewick | 0.03722 | 0.12070 | 0.308 | 0.7578 |
| Pen-y-chain | -0.07125 | 0.13517 | -0.527 | 0.5981 |
| Porthdinllaen | 0.14680 | 0.11112 | 1.321 | 0.1865 |
| Skomer | 0.01944 | 0.11221 | -0.173 | 0.8625 |
| Isles of Scilly (Little Arthur) | 0.27692 | 0.10901 | 2.540 | 0.0111 * |
| **Seagrass cover** formula=glm(seagrass_cover~site, family=quasibinomial) | | | **t-value** |  |
| (Intercept) Criccieth | -4.426 | 1.492 | -2.967 | 0.00332 ** |
| Littlewick | 2.208 | 1.510 | 1.462 | 0.14504 |
| Pen-y-chain | 1.228 | 1.530 | 0.802 | 0.42318 |
| Porthdinllaen | 4.596 | 1.498 | 3.068 | 0.00241 ** |
| Skomer | 3.085 | 1.506 | 2.048 | 0.04172 * |
| **Algae cover**  formula=glm(algae_cover~site, family=quasibinomial) | | |  |  |
| (Intercept) Criccieth | -17.57 | 816.92 | -0.022 | 0.983 |
| Littlewick | 17.36 | 816.92 | 0.021 | 0.983 |
| Pen-y-chain | 16.02 | 816.92 | 0.020 | 0.984 |
| Porthdinllaen | 16.69 | 816.92 | 0.020 | 0.984 |
| Skomer | 15.96 | 816.92 | 0.020 | 0.984 |
| **Seagrass cover ~algae cover** formula=glm(seagrass cover ~algae cover + as.factor (Site), family = quasibinomial) | | |  |  |
| (Intercept) as.factor(Site)Criccieth | -4.4262 | 1.3038 | -3.395 | 0.000808 *** |
| Algae cover | -4.1674 | 0.4684 | -8.898 | < 2e-16 *** |
| as.factor(Site)Littlewick | 3.6014 | 1.3260 | 2.716 | 0.007108 ** |
| as.factor(Site)Pen-y-chain | 1.4919 | 1.3379 | 1.115 | 0.265969 |
| as.factor(Site)Porthdinllaen | 5.7542 | 1.3161 | 4.372 | 1.86e-05 *** |
| as.factor(Site)Skomer | 3.6233 | 1.3179 | 2.749 | 0.006447 ** |

Table A 3. Results from Tukey Pairwise comparisons between seagrass sites for shoot density, number of leaves, max. leaf length and leaf width.

| **Shoot density** | **Estimate** | **Std.Error** | **z-value** | **Pr(>\|z\|)** |
| --- | --- | --- | --- | --- |
| Isles of Scilly - Criccieth | 1.12281 | 0.33264 | 3.375 | 0.00848 ** |
| Littlewick - Criccieth | 0.32158 | 0.36596 | 0.879 | 0.94712 |
| Pen-y-chain - Criccieth | 0.41689 | 0.40103 | 1.04 | 0.89648 |
| Porthdinllaen - Criccieth | 1.40538 | 0.33305 | 4.22 | < 0.001 *** |
| Skomer - Criccieth | 1.11004 | 0.33602 | 3.304 | 0.01093 * |
| Littlewick - Isles of Scilly | -0.80122 | 0.22517 | -3.558 | 0.00454 ** |
| Pen-y-chain - Isles of Scilly | -0.70591 | 0.27857 | -2.534 | 0.10431 |
| Porthdinllaen - Isles of Scilly | 0.28258 | 0.16643 | 1.698 | 0.51154 |
| Skomer - Isles of Scilly | -0.01276 | 0.17229 | -0.074 | 1 |
| Pen-y-chain - Littlewick | 0.09531 | 0.31761 | 0.3 | 0.99964 |
| Porthdinllaen - Littlewick | 1.0838 | 0.22578 | 4.8 | < 0.001 *** |
| Skomer - Littlewick | 0.78846 | 0.23013 | 3.426 | 0.00724 ** |
| Porthdinllaen - Pen-y-chain | 0.98849 | 0.27906 | 3.542 | 0.00488 ** |
| Skomer - Pen-y-chain | 0.69315 | 0.2826 | 2.453 | 0.12698 |
| Skomer - Porthdinllaen | -0.29534 | 0.17308 | -1.706 | 0.50585 |
| **No. leaves** |  |  |  |  |
| Pen-y-chain - Criccieth | -0.07125 | 0.13517 | -0.527 | 0.99451 |
| Littlewick - Criccieth | 0.03722 | 0.1207 | 0.308 | 0.99958 |
| Porthdinllaen - Criccieth | 0.1468 | 0.11112 | 1.321 | 0.75609 |
| Skomer - Criccieth | -0.01944 | 0.11221 | -0.173 | 0.99998 |
| Isles of Scilly - Criccieth | 0.27692 | 0.10901 | 2.54 | 0.10189 |
| Littlewick - Pen-y-chain | 0.10846 | 0.10647 | 1.019 | 0.90335 |
| Porthdinllaen - Pen-y-chain | 0.21805 | 0.09547 | 2.284 | 0.18401 |
| Skomer - Pen-y-chain | 0.05181 | 0.09673 | 0.536 | 0.99408 |
| Isles of Scilly - Pen-y-chain | 0.34817 | 0.093 | 3.744 | 0.00221 ** |
| Porthdinllaen - Littlewick | 0.10959 | 0.07356 | 1.49 | 0.64964 |
| Skomer - Littlewick | -0.05666 | 0.0752 | -0.753 | 0.97225 |
| Isles of Scilly - Littlewick | 0.23971 | 0.07033 | 3.408 | 0.00756 ** |
| Skomer - Porthdinllaen | -0.16624 | 0.05859 | -2.837 | 0.04631 * |
| Isles of Scilly - Porthdinllaen | 0.13012 | 0.0522 | 2.493 | 0.11442 |
| Isles of Scilly - Skomer | 0.29636 | 0.05448 | 5.44 | < 0.001 *** |
| **Leaf width** |  |  |  |  |
| Pen-y-chain - Criccieth | 0.59841 | 0.03065 | 19.526 | <0.001 *** |
| Littlewick - Criccieth | 0.93534 | 0.02749 | 34.023 | <0.001 *** |
| Porthdinllaen - Criccieth | 0.82705 | 0.02531 | 32.678 | <0.001 *** |
| Skomer - Criccieth | 0.78844 | 0.02551 | 30.903 | <0.001 *** |
| Littlewick - Pen-y-chain | 0.33693 | 0.02407 | 13.997 | <0.001 *** |
| Porthdinllaen - Pen-y-chain | 0.22864 | 0.02154 | 10.612 | <0.001 *** |
| Skomer - Pen-y-chain | 0.19003 | 0.02178 | 8.723 | <0.001 *** |
| Porthdinllaen - Littlewick | -0.10829 | 0.01676 | -6.463 | <0.001 *** |
| Skomer - Littlewick | -0.1469 | 0.01706 | -8.61 | <0.001 *** |
| Skomer - Porthdinllaen | -0.03861 | 0.01326 | -2.912 | 0.0271 * |
| **Max. leaf length** |  |  |  |  |
| Isles of Scilly - Criccieth | 1.195614 | 0.067397 | 17.74 | < 0.001 *** |
| Littlewick - Criccieth | 0.859805 | 0.074284 | 11.575 | < 0.001 *** |
| Pen-y-chain - Criccieth | 0.306165 | 0.081254 | 3.768 | 0.00209 ** |
| Porthdinllaen - Criccieth | 0.855574 | 0.068464 | 12.497 | < 0.001 *** |
| Skomer - Criccieth | 0.591973 | 0.068341 | 8.662 | < 0.001 *** |
| Littlewick - Isles of Scilly | -0.33581 | 0.045844 | -7.325 | < 0.001 *** |
| Pen-y-chain - Isles of Scilly | -0.88945 | 0.056442 | -15.759 | < 0.001 *** |
| Porthdinllaen - Isles of Scilly | -0.34004 | 0.03565 | -9.538 | < 0.001 *** |
| Skomer - Isles of Scilly | -0.60364 | 0.035411 | -17.046 | < 0.001 *** |
| Pen-y-chain - Littlewick | -0.55364 | 0.06451 | -8.582 | < 0.001 *** |
| Porthdinllaen - Littlewick | -0.00423 | 0.047399 | -0.089 | 1 |
| Skomer - Littlewick | -0.26783 | 0.04722 | -5.672 | < 0.001 *** |
| Porthdinllaen - Pen-y-chain | 0.549409 | 0.057713 | 9.52 | < 0.001 *** |
| Skomer - Pen-y-chain | 0.285808 | 0.057566 | 4.965 | < 0.001 *** |
| Skomer - Porthdinllaen | -0.2636 | 0.037404 | -7.047 | < 0.001 *** |
| **Seagrass cover** |  |  |  |  |
| Littlewick - Criccieth | 2.2081 | 1.5101 | 1.462 | 0.5448 |
| Pen-y-chain - Criccieth | 1.228 | 1.5305 | 0.802 | 0.9175 |
| Porthdinllaen - Criccieth | 4.5965 | 1.4982 | 3.068 | 0.0142 * |
| Skomer - Criccieth | 3.0846 | 1.5064 | 2.048 | 0.2094 |
| Pen-y-chain - Littlewick | -0.9802 | 0.4141 | -2.367 | 0.1031 |
| Porthdinllaen - Littlewick | 2.3884 | 0.2714 | 8.801 | <0.001 *** |
| Skomer - Littlewick | 0.8765 | 0.3135 | 2.796 | 0.0330 * |
| Porthdinllaen - Pen-y-chain | 3.3685 | 0.3682 | 9.148 | <0.001 *** |
| Skomer - Pen-y-chain | 1.8567 | 0.4003 | 4.639 | <0.001 *** |
| Skomer - Porthdinllaen | -1.5118 | 0.2498 | -6.053 | <0.001 *** |
| **Algae cover** |  |  |  |  |
| Littlewick - Criccieth | 17.35333 | 816.5443 | 0.021 | 1 |
| Pen-y-chain - Criccieth | 16.01885 | 816.5443 | 0.02 | 1 |
| Porthdinllaen - Criccieth | 16.68803 | 816.5443 | 0.02 | 1 |
| Skomer - Criccieth | 15.96194 | 816.5444 | 0.02 | 1 |
| Pen-y-chain - Littlewick | -1.33448 | 0.28625 | -4.662 | < 1e-04 *** |
| Porthdinllaen - Littlewick | -0.6653 | 0.26304 | -2.529 | 0.065188 . |
| Skomer - Littlewick | -1.3914 | 0.34079 | -4.083 | 0.000275 *** |
| Porthdinllaen - Pen-y-chain | 0.66919 | 0.29466 | 2.271 | 0.123824 |
| Skomer - Pen-y-chain | -0.05691 | 0.36576 | -0.156 | 0.999832 |
| Skomer - Porthdinllaen | -0.7261 | 0.34789 | -2.087 | 0.185918 |
| **Epiphytes (binomial)** |  |  |  |  |
| Isles of Scilly - Criccieth | -0.82452 | 0.31333 | -2.632 | 0.08572 . |
| Littlewick - Criccieth | -0.6641 | 0.29225 | -2.272 | 0.19773 |
| Pen-y-chain - Criccieth | -0.24712 | 0.31596 | -0.782 | 0.9691 |
| Porthdinllaen - Criccieth | -1.00852 | 0.26815 | -3.761 | 0.00222 ** |
| Skomer - Criccieth | -0.64946 | 0.26455 | -2.455 | 0.13204 |
| Littlewick - Isles of Scilly | 0.16042 | 0.26777 | 0.599 | 0.99063 |
| Pen-y-chain - Isles of Scilly | 0.5774 | 0.29347 | 1.968 | 0.35053 |
| Porthdinllaen - Isles of Scilly | -0.18399 | 0.24124 | -0.763 | 0.9723 |
| Skomer - Isles of Scilly | 0.17506 | 0.23724 | 0.738 | 0.97602 |
| Pen-y-chain - Littlewick | 0.41698 | 0.27085 | 1.54 | 0.62797 |
| Porthdinllaen - Littlewick | -0.34442 | 0.21315 | -1.616 | 0.57708 |
| Skomer - Littlewick | 0.01464 | 0.20861 | 0.07 | 1 |
| Porthdinllaen - Pen-y-chain | -0.7614 | 0.24465 | -3.112 | 0.02169 * |
| Skomer - Pen-y-chain | -0.40234 | 0.2407 | -1.672 | 0.53937 |
| Skomer - Porthdinllaen | 0.35906 | 0.17325 | 2.073 | 0.29189 |
| **Wasting** |  |  |  |  |
| Isles of Scilly - Criccieth | -1.2557 | 0.9513 | -1.32 | 0.753 |
| Littlewick - Criccieth | 1.1674 | 0.6339 | 1.842 | 0.41 |
| Pen-y-chain - Criccieth | -0.511 | 0.9089 | -0.562 | 0.992 |
| Porthdinllaen - Criccieth | -0.6738 | 0.6911 | -0.975 | 0.917 |
| Skomer - Criccieth | -0.2895 | 0.6614 | -0.438 | 0.998 |
| Littlewick - Isles of Scilly | 2.4231 | 0.7707 | 3.144 | 0.018 * |
| Pen-y-chain - Isles of Scilly | 0.7447 | 1.0091 | 0.738 | 0.974 |
| Porthdinllaen - Isles of Scilly | 0.5819 | 0.8184 | 0.711 | 0.978 |
| Skomer - Isles of Scilly | 0.9661 | 0.7935 | 1.218 | 0.811 |
| Pen-y-chain - Littlewick | -1.6783 | 0.7176 | -2.339 | 0.161 |
| Porthdinllaen - Littlewick | -1.8411 | 0.4082 | -4.511 | <0.001 *** |
| Skomer - Littlewick | -1.4569 | 0.3555 | -4.098 | <0.001 *** |
| Porthdinllaen - Pen-y-chain | -0.1628 | 0.7686 | -0.212 | 1 |
| Skomer - Pen-y-chain | 0.2214 | 0.742 | 0.298 | 1 |
| Skomer - Porthdinllaen | 0.3842 | 0.4497 | 0.854 | 0.951 |

Table A.4. Results from the Principal Component Analysis carried out using shoot data (PCA1), quadrat level data to include shoot density and no. leaves per shoot (PCA2) and nutrient and stable isotope meadow data (PCA3) for sites around Wales. Bold values show significant levels of eigenvalues (above 1 for principal component, and eigenfactors or variable coefficients ≤−0.3, or ≥ 0.3).

| **PCA1 – Shoot data** | **PC1** | **PC2** | **PC3** |
| --- | --- | --- | --- |
| *Summary Values* |  |  |  |
| Eigenvalues | **2.15** | **1.18** | 0.48 |
| Percent variation | 53.7 | 29.6 | 11.9 |
| Cumulative percent variation | 53.7 | 83.3 | 95.3 |
| *Seagrass variables* |  |  |  |
| Leaf length | **0.591** | **-0.320** | 0.288 |
| Leaf width | **0.589** | **-0.359** | 0.092 |
| Epiphyte | 0.254 | **0.769** | **0.577** |
| Wasting | **0.489** | **0.420** | **-0.758** |
|  |  |  |  |
| **PCA2 – Quadrat data** | **PC1** | **PC2** | **PC3** |
| *Summary Values* |  |  |  |
| Eigenvalues | **2.37** | **1.52** | 0.93 |
| Percent variation | 39.5 | 25.4 | 15.5 |
| Cumulative percent variation | 39.5 | 64.9 | 80.5 |
| *Seagrass variables* |  |  |  |
| Density | 0.291 | -0.294 | **-0.810** |
| Leaf length | **0.607** | 0.111 | -0.078 |
| Leaf width | **0.606** | 0.077 | 0.117 |
| Epiphyte | -0.216 | **0.465** | **-0.475** |
| Wasting | 0.282 | **0.661** | 0.171 |
| Leaves per shoot | 0.232 | **-0.492** | 0.265 |
|  |  |  |  |
| **PCA3 – Nutrient data** |  |  |  |
| *Summary Values* | PC1 | PC2 | PC3 |
| Eigenvalues | **2.54** | **1.99** | **1.12** |
| Percent variation | 42.3 | 33.2 | 18.6 |
| Cumulative percent variation | 42.3 | 75.5 | 94.1 |
| *Seagrass variables* |  |  |  |
| % N | **-0.560** | 0.217 | -0.140 |
| % P | 0.040 | **0.683** | 0.078 |
| C:N | **0.567** | 0.170 | -0.241 |
| δ^15^N | 0.088 | **0.642** | **0.303** |
| δ^13^C | **0.596** | -0.104 | 0.075 |
| Density | 0.019 | 0.186 | **-0.905** |

Table A.5. Analysis of Deviance table showing results of the likelihood ratios test for comparing GLM models with and without ‘Year’ to assess significance of test for shoot density and leaf lengths from long-term monitoring data.

| Skomer | | | | | | |
| --- | --- | --- | --- | --- | --- | --- |
| Model 1: Z.marina_density ~ 1 | | | | | | |
| Model 2: Z.marina_density ~ as.factor(Year) | | | | | | |
|  | Resid. Df | Resid. Dev | Df | Deviance | F | Pr(>F) |
| 1 | 1992 | 51997 |  |  |  |  |
| 2 | 1986 | 47325 | 6 | 4672 | 36.774 | **< 2.2e-16 ***** |
| Isles of Scilly - Little Arthur | | | | | | |
| Model 1: density_m2 ~ 1 | | | | | | |
| Model 2: density_m2 ~ as.factor(Year) | | | | | | |
|  | Resid. Df | Resid. Dev | Df | Deviance | F | Pr(>F) |
| 1 | 516 | 33303 |  |  |  |  |
| 2 | 495 | 29204 | 21 | 4099.2 | 3.7908 | **5.013e-08 ***** |
| Model 1: max_length ~ 1 | | | | | | |
| Model 2: max_length ~ as.factor(year) | | | | | | |
|  | Resid. Df | Resid. Dev | Df | Deviance | F | Pr(>F) |
| 1 | 5166 | 680.75 |  |  |  |  |
| 2 | 5145 | 583.63 | 21 | 97.118 | 55.368 | **< 2.2e-16 ***** |
| Model 1: av_inf ~ 1 | | | | | | |
| Model 2: av_inf ~ as.factor(year) | | | | | | |
|  | Resid. Df | Resid. Dev | Df | Deviance | Pr(>Chi) |  |
| 1 | 5207 | 240.85 |  |  |  |  |
| 2 | 5186 | 196.69 | 21 | 44.152 | **0.002234 **** | |
| Model 1: prop_epiphytes ~ 1 | | |  |  |  |  |
| Model 2: prop_epiphytes ~ Year | | |  |  |  |  |
|  | Resid. Df | Resid. Dev | Df | Deviance | Pr(>Chi) |  |
| 1 | 5207 | 588.53 |  |  |  |  |
| 2 | 5186 | 359.5 | 21 | 229.03 | **< 2.2e-16 ***** | |
| Porthdinllaen | | | | | | |
| Model 1: Z.marina_density ~ 1 | | | | | | |
| Model 2: Z.marina_density ~ as.factor(Year) | | | | | | |
|  | Resid. Df | Resid. Dev | Df | Deviance | F | Pr(>F) |
| 1 | 584 | 22633 |  |  |  |  |
| 2 | 580 | 22507 | 4 | 125.78 | 0.9984 | 0.4078 |
| Model 1: leaf_length ~ 1 | | | | | | |
| Model 2: leaf_length ~ as.factor(Year) | | | | | | |
|  | Resid. Df | Resid. Dev | Df | Deviance | F | Pr(>F) |
| 1 | 1378 | 357.09 |  |  |  |  |
| 2 | 1374 | 351.57 | 4 | 5.5175 | 5.7486 | **0.0001375 ***** |
| Littlewick | | | | | | |
| Model 1: Z.marina_density ~ as.factor(Year) | | | | | | |
| Model 2: Z.marina_density ~ 1 | | | | | | |
|  | Resid. Df | Resid. Dev | Df | Deviance | F | Pr(>F) |
| 1 | 906 | 33543 |  |  |  |  |
| 2 | 911 | 44976 | -5 | -11433 | 71.322 | **< 2.2e-16 ***** |
| Model 1: leaf_length ~ 1 | | | | | | |
| Model 2: leaf_length ~ as.factor(Year) | | | | | | |
|  | Resid. Df | Resid. Dev | Df | Deviance | F | Pr(>F) |
| 1 | 7424 | 1990.9 |  |  |  |  |
| 2 | 7419 | 1802.8 | 5 | 188.17 | 181.32 | **< 2.2e-16 ***** |
| Model 1: prop_wasting ~ 1 | | | | | | |
| Model 2: prop_wasting ~ as.factor(Year) | | | | | | |
|  | Resid. Df | Resid. Dev | Df | Deviance | Pr(>Chi) |  |
| 1 | 4051 | 1016.51 |  |  |  |  |
| 2 | 4048 | 858.51 | 3 | 158 | **< 2.2e-16 ***** | |
| Model 1: prop_epiphytes ~ 1 | | | | | | |
| Model 2: prop_epiphytes ~ as.factor(Year) | | | | | | |
|  | Resid. Df | Resid. Dev | Df | Deviance | Pr(>Chi) |  |
| 1 | 4051 | 1904.1 |  |  |  |  |
| 2 | 4048 | 1370.2 | 3 | 533.86 | **< 2.2e-16 ***** | |

Table A.6. Summary output results from GLM for shoot count data (shoot density per m^2^) taken from long-term monitoring data for Littlewick, Skomer, Porthdinllaen and the Isles of Scilly.

| **Coefficients - Year** | **Estimate** | **Std. Error** | **t value** | **Pr(>\|t\|)** |
| --- | --- | --- | --- | --- |
| **Littlewick** | | | | |
| (Intercept) 1986 | 4.946 | 0.068 | 72.507 | < 2e-16 *** |
| as.factor(Year)1999 | 0.005 | 0.079 | 0.070 | 0.944247 |
| as.factor(Year)2008 | -0.292 | 0.079 | -3.712 | 0.000219 *** |
| as.factor(Year)2012 | -0.875 | 0.080 | -10.926 | < 2e-16 *** |
| as.factor(Year)2016 | -0.787 | 0.176 | -4.469 | 8.84e-06 *** |
| as.factor(Year)2018 | -0.849 | 0.087 | -9.779 | < 2e-16 *** |
| **Skomer** |  | | | |
| (Intercept) 1997 | 3.761 | 0.045 | 83.545 | < 2e-16 *** |
| as.factor(Year)2002 | 0.283 | 0.071 | 3.965 | 7.61e-05 *** |
| as.factor(Year)2006 | 0.195 | 0.056 | 3.473 | 0.000525 *** |
| as.factor(Year)2010 | -0.082 | 0.057 | -1.451 | 0.146938 |
| as.factor(Year)2014 | -0.173 | 0.059 | -2.914 | 0.003605 ** |
| as.factor(Year)2016 | 1.186 | 0.098 | 12.141 | < 2e-16 *** |
| as.factor(Year)2018 | 0.172 | 0.055 | 3.129 | 0.001782 ** |
| **Porthdinllaen** | formula=glm(shoot_density~year, family = quasipoisson) | | | |
| (Intercept) 2015 | 3.841 | 0.083 | 46.468 | <2e-16 *** |
| as.factor(Year)2016 | -0.091 | 0.106 | -0.855 | 0.393 |
| as.factor(Year)2017 | -0.128 | 0.121 | -1.062 | 0.289 |
| as.factor(Year)2018 | -0.075 | 0.111 | -0.671 | 0.503 |
| as.factor(Year)2019 | 0.080 | 0.117 | 0.682 | 0.496 |
| **Isles of Scilly** | formula=glm(shoot_density~year, family = quasipoisson) | | | |
| (Intercept)1998 | 4.88643 | 0.12469 | 39.189 | < 2e-16 *** |
| as.factor(Year)1999 | 0.17261 | 0.1692 | 1.02 | 0.30816 |
| as.factor(Year)2000 | 0.15622 | 0.16984 | 0.92 | 0.35813 |
| as.factor(Year)2001 | 0.05635 | 0.1739 | 0.324 | 0.74604 |
| as.factor(Year)2002 | 0.51152 | 0.16323 | 3.134 | 0.00183 ** |
| as.factor(Year)2003 | 0.66124 | 0.15354 | 4.307 | 2e-05 *** |
| as.factor(Year)2004 | 0.42054 | 0.16323 | 2.576 | 0.01027 * |
| as.factor(Year)2005 | 0.01439 | 0.17571 | 0.082 | 0.93477 |
| as.factor(Year)2006 | 0.04013 | 0.18256 | 0.22 | 0.8261 |
| as.factor(Year)2007 | 0.37106 | 0.16347 | 2.27 | 0.02364 * |
| as.factor(Year)2008 | 0.30919 | 0.16419 | 1.883 | 0.06028 . |
| as.factor(Year)2009 | 0.15914 | 0.17135 | 0.929 | 0.35346 |
| as.factor(Year)2010 | 0.24431 | 0.17333 | 1.409 | 0.15932 |
| as.factor(Year)2011 | -0.12537 | 0.18629 | -0.673 | 0.50126 |
| as.factor(Year)2012 | 0.03326 | 0.17489 | 0.19 | 0.84926 |
| as.factor(Year)2013 | 0.24185 | 0.17152 | 1.41 | 0.15915 |
| as.factor(Year)2014 | -0.19203 | 0.18754 | -1.024 | 0.30636 |
| as.factor(Year)2015 | -0.22073 | 0.18691 | -1.181 | 0.23819 |
| as.factor(Year)2016 | 0.07367 | 0.17677 | 0.417 | 0.67702 |
| as.factor(Year)2017 | 0.26106 | 0.17469 | 1.494 | 0.1357 |
| as.factor(Year)2018 | 0.1097 | 0.1717 | 0.639 | 0.52317 |
| as.factor(Year)2019 | 0.40616 | 0.16697 | 2.433 | 0.01535 * |

Table A.7. Tukey pairwise comparison results for GLM of shoot density between years at sites where Year was found to be a significant factor.

| **Littlewick density** |  |  |  |  |
| --- | --- | --- | --- | --- |
| 1999 - 1986 | 0.005492 | 0.078517 | 0.07 | 1 |
| 2008 - 1986 | -0.29214 | 0.078711 | -3.712 | 0.00243 ** |
| 2012 - 1986 | -0.87487 | 0.080072 | -10.926 | < 0.001 *** |
| 2016 - 1986 | -0.78712 | 0.17612 | -4.469 | < 0.001 *** |
| 2018 - 1986 | -0.84878 | 0.086794 | -9.779 | < 0.001 *** |
| 2008 - 1999 | -0.29763 | 0.055263 | -5.386 | < 0.001 *** |
| 2012 - 1999 | -0.88036 | 0.057185 | -15.395 | < 0.001 *** |
| 2016 - 1999 | -0.79262 | 0.166964 | -4.747 | < 0.001 *** |
| 2018 - 1999 | -0.85428 | 0.06627 | -12.891 | < 0.001 *** |
| 2012 - 2008 | -0.58272 | 0.057452 | -10.143 | < 0.001 *** |
| 2016 - 2008 | -0.49498 | 0.167055 | -2.963 | 0.03128 * |
| 2018 - 2008 | -0.55664 | 0.0665 | -8.371 | < 0.001 *** |
| 2016 - 2012 | 0.087742 | 0.167701 | 0.523 | 0.9946 |
| 2018 - 2012 | 0.026083 | 0.068106 | 0.383 | 0.99878 |
| 2018 - 2016 | -0.06166 | 0.171012 | -0.361 | 0.99909 |
| **Skomer density** |  |  |  |  |
| 2002 - 1997 | 0.28296 | 0.07137 | 3.965 | 0.00124 ** |
| 2006 - 1997 | 0.19549 | 0.05628 | 3.473 | 0.00858 ** |
| 2010 - 1997 | -0.08245 | 0.05682 | -1.451 | 0.76091 |
| 2014 - 1997 | -0.1733 | 0.05947 | -2.914 | 0.05086 . |
| 2016 - 1997 | 1.18624 | 0.0977 | 12.141 | < 0.001 *** |
| 2018 - 1997 | 0.1717 | 0.05488 | 3.129 | 0.02653 * |
| 2006 - 2002 | -0.08747 | 0.06487 | -1.348 | 0.81816 |
| 2010 - 2002 | -0.36541 | 0.06534 | -5.593 | < 0.001 *** |
| 2014 - 2002 | -0.45626 | 0.06765 | -6.744 | < 0.001 *** |
| 2016 - 2002 | 0.90327 | 0.10289 | 8.779 | < 0.001 *** |
| 2018 - 2002 | -0.11126 | 0.06366 | -1.748 | 0.56627 |
| 2010 - 2006 | -0.27793 | 0.0484 | -5.742 | < 0.001 *** |
| 2014 - 2006 | -0.36879 | 0.05148 | -7.163 | < 0.001 *** |
| 2016 - 2006 | 0.99075 | 0.09306 | 10.646 | < 0.001 *** |
| 2018 - 2006 | -0.02379 | 0.04611 | -0.516 | 0.99851 |
| 2014 - 2010 | -0.09085 | 0.05207 | -1.745 | 0.56838 |
| 2016 - 2010 | 1.26868 | 0.09339 | 13.585 | < 0.001 *** |
| 2018 - 2010 | 0.25414 | 0.04677 | 5.434 | < 0.001 *** |
| 2016 - 2014 | 1.35954 | 0.09502 | 14.308 | < 0.001 *** |
| 2018 - 2014 | 0.345 | 0.04995 | 6.907 | < 0.001 *** |
| 2018 - 2016 | -1.01454 | 0.09222 | -11.001 | < 0.001 *** |
| **Isles of Scilly (Little Arthur) Density -** only significant rows only shown | | | | |
| 2003 - 1998 | 0.661243 | 0.153535 | 4.307 | <0.01 ** |
| 2003 - 2001 | 0.60489 | 0.150736 | 4.013 | 0.0106 * |
| 2011 - 2002 | -0.63689 | 0.173937 | -3.662 | 0.0383 * |
| 2014 - 2002 | -0.70355 | 0.175275 | -4.014 | 0.0105 * |
| 2015 - 2002 | -0.73225 | 0.174599 | -4.194 | <0.01 ** |
| 2005 - 2003 | -0.64685 | 0.15281 | -4.233 | <0.01 ** |
| 2006 - 2003 | -0.62111 | 0.160644 | -3.866 | 0.0198 * |
| 2011 - 2003 | -0.78662 | 0.164871 | -4.771 | <0.01 *** |
| 2012 - 2003 | -0.62799 | 0.15187 | -4.135 | <0.01 ** |
| 2014 - 2003 | -0.85327 | 0.166281 | -5.132 | <0.01 *** |
| 2015 - 2003 | -0.88197 | 0.165569 | -5.327 | <0.01 *** |
| 2016 - 2003 | -0.58757 | 0.154029 | -3.815 | 0.0229 * |
| 2018 - 2003 | -0.55154 | 0.148182 | -3.722 | 0.0318 * |
| 2015 - 2004 | -0.64128 | 0.174599 | -3.673 | 0.0372 * |

Table A.8. Summary output results from GLM for *Z. marina* leaf lengths taken from long-term monitoring data available for Littlewick, Porthdinllaen and the Isles of Scilly.

| **Littlewick** | **Estimate** | **Std. Error** | **t value** | **Pr(>\|t\|)** |
| --- | --- | --- | --- | --- |
| (Intercept)1986 | 5.810 | 0.008 | 756.222 | <2e-16 *** |
| as.factor(Year)1999 | 0.183 | 0.014 | 12.453 | <2e-16 *** |
| as.factor(Year)2008 | 0.215 | 0.080 | 2.695 | 0.007 ** |
| as.factor(Year)2012 | -0.336 | 0.015 | -21.960 | <2e-16 *** |
| as.factor(Year)2016 | 0.044 | 0.031 | 1.401 | 0.161 |
| as.factor(Year)2018 | 0.112 | 0.015 | 7.448 | 1.05e-13*** |
| **Porthdinllaen** |  |  |  |  |
| (Intercept) 2015 | 5.512 | 0.034 | 164.229 | < 2e-16 *** |
| as.factor(Year)2016 | -0.062 | 0.042 | -1.480 | 0.139 |
| as.factor(Year)2017 | -0.094 | 0.046 | -2.030 | 0.0425 * |
| as.factor(Year)2018 | -0.198 | 0.044 | -4.512 | 6.96e-06 *** |
| as.factor(Year)2019 | -0.078 | 0.046 | -1.677 | 0.0938 . |
| **Isles of Scilly** | Formula=glm(leaf_length~as.factor(year), family=Gamma(link=”log”)) | | | |
| (Intercept)1998 | 6.755 | 0.02009 | 336.257 | < 2e-16 *** |
| as.factor(year)1999 | -0.08318 | 0.02726 | -3.052 | 0.002289 ** |
| as.factor(year)2000 | 0.03193 | 0.02736 | 1.167 | 0.243318 |
| as.factor(year)2001 | -0.2105 | 0.02802 | -7.512 | 6.82e-14 *** |
| as.factor(year)2002 | -0.00212 | 0.0263 | -0.081 | 0.935789 |
| as.factor(year)2003 | -0.139 | 0.02473 | -5.618 | 2.03e-08 *** |
| as.factor(year)2004 | -0.2101 | 0.0263 | -7.988 | 1.68e-15 *** |
| as.factor(year)2005 | 0.05751 | 0.02831 | 2.032 | 0.042226 * |
| as.factor(year)2006 | 0.1086 | 0.02941 | 3.691 | 0.000226 *** |
| as.factor(year)2007 | 5.81E-05 | 0.02633 | 0.002 | 0.998241 |
| as.factor(year)2008 | -0.2442 | 0.02645 | -9.233 | < 2e-16 *** |
| as.factor(year)2009 | 0.1473 | 0.0276 | 5.337 | 9.84e-08 *** |
| as.factor(year)2010 | 0.001576 | 0.02792 | 0.056 | 0.954992 |
| as.factor(year)2011 | 0.1395 | 0.03001 | 4.647 | 3.45e-06 *** |
| as.factor(year)2012 | -0.1677 | 0.02817 | -5.952 | 2.83e-09 *** |
| as.factor(year)2013 | -0.1343 | 0.02763 | -4.859 | 1.21e-06 *** |
| as.factor(year)2014 | -0.473 | 0.03021 | -15.655 | < 2e-16 *** |
| as.factor(year)2015 | -0.1262 | 0.03011 | -4.19 | 2.83e-05 *** |
| as.factor(year)2016 | -0.3078 | 0.02848 | -10.807 | < 2e-16 *** |
| as.factor(year)2017 | 0.007971 | 0.02814 | 0.283 | 0.777008 |
| as.factor(year)2018 | -0.05338 | 0.02766 | -1.93 | 0.053699 . |
| as.factor(year)2019 | -0.1507 | 0.0269 | -5.601 | 2.24e-08 *** |

Table A.9. Summary output results from GLM for *Z. marina* leaf condition taken from long-term monitoring data available for Littlewick, and the Isles of Scilly.

| **Littlewick - Epiphytes** | **Estimate** | **Std. Error** | **z value** | **Pr(>\|t\|)** |
| --- | --- | --- | --- | --- |
| (Intercept)1998 | -1.01526 | 0.03595 | -28.24 | < 2e-16 *** |
| as.factor(Year)2012 | -2.62373 | 0.17877 | -14.68 | < 2e-16 *** |
| as.factor(Year)2016 | -0.65597 | 0.13333 | -4.92 | 8.65e-07 *** |
| as.factor(Year)2018 | -0.78291 | 0.07364 | -10.63 | < 2e-16 *** |
| **Littlewick - Wasting** |  |  |  |  |
| (Intercept)1998 | -6.1231 | 0.5782 | -10.591 | < 2e-16 *** |
| as.factor(Year)2012 | 3.1441 | 0.5914 | 5.317 | 1.06e-07 *** |
| as.factor(Year)2016 | 4.1164 | 0.599 | 6.872 | 6.35e-12 *** |
| as.factor(Year)2018 | 3.5591 | 0.5866 | 6.067 | 1.30e-09 *** |
| **Isles of Scilly -Epiphytes** |  |  |  |  |
| (Intercept) 1998 | -1.66631 | 0.1423 | -11.71 | < 2e-16 *** |
| as.factor(year) 1999 | -1.09483 | 0.28367 | -3.86 | 0.000114 *** |
| as.factor(year) 2000 | 0.05128 | 0.19208 | 0.267 | 0.789503 |
| as.factor(year) 2001 | -0.45213 | 0.23166 | -1.952 | 0.050969 . |
| as.factor(year) 2002 | -0.46366 | 0.21405 | -2.166 | 0.030299 * |
| as.factor(year) 2003 | 0.40713 | 0.16276 | 2.501 | 0.012373 * |
| as.factor(year) 2004 | 0.50801 | 0.16652 | 3.051 | 0.002283 ** |
| as.factor(year) 2005 | 0.22729 | 0.18727 | 1.214 | 0.224861 |
| as.factor(year) 2006 | 0.33966 | 0.18808 | 1.806 | 0.070933 . |
| as.factor(year) 2007 | -0.52363 | 0.21819 | -2.4 | 0.016403 * |
| as.factor(year) 2008 | -0.41848 | 0.21266 | -1.968 | 0.049083 * |
| as.factor(year) 2009 | 0.05365 | 0.19361 | 0.277 | 0.781717 |
| as.factor(year) 2010 | -0.01333 | 0.19976 | -0.067 | 0.946795 |
| as.factor(year) 2011 | 0.44217 | 0.18562 | 2.382 | 0.017215 * |
| as.factor(year) 2012 | 0.76845 | 0.16435 | 4.676 | 2.93e-06 *** |
| as.factor(year) 2013 | -0.02492 | 0.19831 | -0.126 | 0.899999 |
| as.factor(year) 2014 | -0.32655 | 0.23984 | -1.362 | 0.173343 |
| as.factor(year) 2015 | 0.45309 | 0.18459 | 2.455 | 0.014106 * |
| as.factor(year) 2016 | -0.35887 | 0.22809 | -1.573 | 0.115631 |
| as.factor(year) 2017 | 0.54447 | 0.17234 | 3.159 | 0.001582 ** |
| as.factor(year) 2018 | 0.16926 | 0.18761 | 0.902 | 0.36695 |
| as.factor(year) 2019 | 0.26257 | 0.17892 | 1.468 | 0.142231 |
| **Isles of Scilly -Wasting** |  |  |  |  |
| (Intercept) 1998 | -1.9705 | 0.17066 | -11.547 | < 2e-16 *** |
| as.factor(year) 1999 | -0.11344 | 0.24026 | -0.472 | 0.63683 |
| as.factor(year) 2000 | 0.15518 | 0.22445 | 0.691 | 0.48933 |
| as.factor(year) 2001 | 0.62283 | 0.20526 | 3.034 | 0.00241 ** |
| as.factor(year) 2002 | 0.15777 | 0.21634 | 0.729 | 0.46585 |
| as.factor(year) 2003 | 0.32436 | 0.19872 | 1.632 | 0.10262 |
| as.factor(year) 2004 | 0.1541 | 0.21624 | 0.713 | 0.47607 |
| as.factor(year) 2005 | 0.24737 | 0.22435 | 1.103 | 0.27018 |
| as.factor(year) 2006 | 0.36706 | 0.22543 | 1.628 | 0.10347 |
| as.factor(year) 2007 | -0.14598 | 0.23303 | -0.626 | 0.53104 |
| as.factor(year) 2008 | 0.37476 | 0.20753 | 1.806 | 0.07094 . |
| as.factor(year) 2009 | -0.02865 | 0.23773 | -0.12 | 0.90409 |
| as.factor(year) 2010 | 0.38892 | 0.21569 | 1.803 | 0.07137 . |
| as.factor(year) 2011 | 0.38648 | 0.22815 | 1.694 | 0.09026 . |
| as.factor(year) 2012 | 0.33056 | 0.22011 | 1.502 | 0.13316 |
| as.factor(year) 2013 | 0.37774 | 0.2145 | 1.761 | 0.07823 . |
| as.factor(year) 2014 | 0.29436 | 0.23384 | 1.259 | 0.20811 |
| as.factor(year) 2015 | 0.595 | 0.21545 | 2.762 | 0.00575 ** |
| as.factor(year) 2016 | 0.24279 | 0.22689 | 1.07 | 0.28459 |
| as.factor(year) 2017 | 0.34254 | 0.21867 | 1.566 | 0.11724 |
| as.factor(year) 2018 | 0.03039 | 0.23412 | 0.13 | 0.89673 |
| as.factor(year) 2019 | 0.05771 | 0.22636 | 0.255 | 0.79875 |

Table A.10. Tukey pairwise comparison results for GLM of leaf length and leaf condition (wasting disease and epiphyte cover) between years at sites where Year was found to be a significant factor. Only the significant pairwise results are displayed for the Isles of Scilly due to the large dataset.

| **Site/variable** | **Estimate** | **Std. Error** | **z value** | **Pr(>\|z\|)** |
| --- | --- | --- | --- | --- |
| **Porthdinllaen length** | | | | |
| 2016 - 2015 | -0.06181 | 0.04177 | -1.48 | 0.57392 |
| 2017 - 2015 | -0.09389 | 0.04625 | -2.03 | 0.25005 |
| 2018 - 2015 | -0.19751 | 0.04377 | -4.512 | < 0.001 *** |
| 2019 - 2015 | -0.07755 | 0.04625 | -1.677 | 0.44657 |
| 2017 - 2016 | -0.03208 | 0.04038 | -0.794 | 0.93188 |
| 2018 - 2016 | -0.1357 | 0.03752 | -3.617 | 0.00281 ** |
| 2019 - 2016 | -0.01574 | 0.04038 | -0.39 | 0.99508 |
| 2018 - 2017 | -0.10362 | 0.04245 | -2.441 | 0.10334 |
| 2019 - 2017 | 0.01633 | 0.045 | 0.363 | 0.99627 |
| 2019 - 2018 | 0.11996 | 0.04245 | 2.826 | 0.03768 * |
| **Littlewick length** | | | | |
| 1999 - 1986 | 0.1826 | 0.01466 | 12.453 | <0.001 *** |
| 2008 - 1986 | 0.21482 | 0.0797 | 2.695 | 0.0609 . |
| 2012 - 1986 | -0.33624 | 0.01531 | -21.96 | <0.001 *** |
| 2016 - 1986 | 0.04406 | 0.03145 | 1.401 | 0.6869 |
| 2018 - 1986 | 0.11157 | 0.01498 | 7.448 | <0.001 *** |
| 2008 - 1999 | 0.03222 | 0.08026 | 0.401 | 0.9983 |
| 2012 - 1999 | -0.51884 | 0.01801 | -28.815 | <0.001 *** |
| 2016 - 1999 | -0.13853 | 0.03285 | -4.217 | <0.001 *** |
| 2018 - 1999 | -0.07103 | 0.01772 | -4.007 | <0.001 *** |
| 2012 - 2008 | -0.55106 | 0.08038 | -6.855 | <0.001 *** |
| 2016 - 2008 | -0.17076 | 0.08495 | -2.01 | 0.293 |
| 2018 - 2008 | -0.10325 | 0.08032 | -1.285 | 0.7597 |
| 2016 - 2012 | 0.3803 | 0.03314 | 11.475 | <0.001 *** |
| 2018 - 2012 | 0.44781 | 0.01826 | 24.518 | <0.001 *** |
| 2018 - 2016 | 0.06751 | 0.03299 | 2.046 | 0.2738 |
| **Littlwick-wasting** | | | | |
| 2012 - 1999 | 3.1441 | 0.5914 | 5.317 | <0.001 *** |
| 2016 - 1999 | 4.1164 | 0.599 | 6.872 | <0.001 *** |
| 2018 - 1999 | 3.5591 | 0.5866 | 6.067 | <0.001 *** |
| 2016 - 2012 | 0.9723 | 0.2001 | 4.859 | <0.001 *** |
| 2018 - 2012 | 0.4149 | 0.159 | 2.61 | 0.0375 * |
| 2018 - 2016 | -0.5573 | 0.1855 | -3.005 | 0.0117 * |
| **Littlwick-epiphytes** | | | | |
| 2012 - 1999 | -2.62373 | 0.17877 | -14.676 | <1e-04 *** |
| 2016 - 1999 | -0.65597 | 0.13333 | -4.92 | <1e-04 *** |
| 2018 - 1999 | -0.78291 | 0.07364 | -10.632 | <1e-04 *** |
| 2016 - 2012 | 1.96775 | 0.21714 | 9.062 | <1e-04 *** |
| 2018 - 2012 | 1.84082 | 0.18654 | 9.868 | <1e-04 *** |
| 2018 - 2016 | -0.12693 | 0.14357 | -0.884 | 0.797 |
| **Scilly lengths** | | | | |
| 2001 - 1998 | -0.2105 | 0.02802 | -7.512 | <0.01 *** |
| 2003 - 1998 | -0.139 | 0.02473 | -5.618 | <0.01 *** |
| 2004 - 1998 | -0.2101 | 0.0263 | -7.988 | <0.01 *** |
| 2006 - 1998 | 0.1086 | 0.02941 | 3.691 | 0.0357 * |
| 2008 - 1998 | -0.2442 | 0.02645 | -9.233 | <0.01 *** |
| 2009 - 1998 | 0.1473 | 0.0276 | 5.337 | <0.01 *** |
| 2011 - 1998 | 0.1395 | 0.03001 | 4.647 | <0.01 *** |
| 2012 - 1998 | -0.1677 | 0.02817 | -5.952 | <0.01 *** |
| 2013 - 1998 | -0.1343 | 0.02763 | -4.859 | <0.01 *** |
| 2014 - 1998 | -0.473 | 0.03021 | -15.655 | <0.01 *** |
| 2015 - 1998 | -0.1262 | 0.03011 | -4.19 | <0.01 ** |
| 2016 - 1998 | -0.3078 | 0.02848 | -10.807 | <0.01 *** |
| 2019 - 1998 | -0.1507 | 0.0269 | -5.601 | <0.01 *** |
| 2000 - 1999 | 0.1151 | 0.02617 | 4.399 | <0.01 ** |
| 2001 - 1999 | -0.1273 | 0.02685 | -4.74 | <0.01 *** |
| 2004 - 1999 | -0.1269 | 0.02505 | -5.065 | <0.01 *** |
| 2005 - 1999 | 0.1407 | 0.02715 | 5.182 | <0.01 *** |
| 2006 - 1999 | 0.1917 | 0.0283 | 6.775 | <0.01 *** |
| 2008 - 1999 | -0.161 | 0.02521 | -6.387 | <0.01 *** |
| 2009 - 1999 | 0.2305 | 0.02642 | 8.725 | <0.01 *** |
| 2011 - 1999 | 0.2226 | 0.02893 | 7.697 | <0.01 *** |
| 2014 - 1999 | -0.3898 | 0.02913 | -13.379 | <0.01 *** |
| 2016 - 1999 | -0.2246 | 0.02733 | -8.217 | <0.01 *** |
| 2001 - 2000 | -0.2424 | 0.02695 | -8.992 | <0.01 *** |
| 2003 - 2000 | -0.1709 | 0.02353 | -7.264 | <0.01 *** |
| 2004 - 2000 | -0.242 | 0.02516 | -9.617 | <0.01 *** |
| 2008 - 2000 | -0.2762 | 0.02532 | -10.905 | <0.01 *** |
| 2009 - 2000 | 0.1154 | 0.02653 | 4.351 | <0.01 ** |
| 2011 - 2000 | 0.1075 | 0.02902 | 3.705 | 0.0333 * |
| 2012 - 2000 | -0.1996 | 0.02712 | -7.361 | <0.01 *** |
| 2013 - 2000 | -0.1662 | 0.02656 | -6.258 | <0.01 *** |
| 2014 - 2000 | -0.5049 | 0.02923 | -17.273 | <0.01 *** |
| 2015 - 2000 | -0.1581 | 0.02913 | -5.428 | <0.01 *** |
| 2016 - 2000 | -0.3397 | 0.02743 | -12.382 | <0.01 *** |
| 2019 - 2000 | -0.1826 | 0.02579 | -7.08 | <0.01 *** |
| 2002 - 2001 | 0.2083 | 0.02587 | 8.052 | <0.01 *** |
| 2005 - 2001 | 0.268 | 0.02791 | 9.6 | <0.01 *** |
| 2006 - 2001 | 0.319 | 0.02903 | 10.988 | <0.01 *** |
| 2007 - 2001 | 0.2105 | 0.02591 | 8.124 | <0.01 *** |
| 2009 - 2001 | 0.3578 | 0.0272 | 13.154 | <0.01 *** |
| 2010 - 2001 | 0.212 | 0.02753 | 7.703 | <0.01 *** |
| 2011 - 2001 | 0.3499 | 0.02964 | 11.805 | <0.01 *** |
| 2014 - 2001 | -0.2625 | 0.02984 | -8.796 | <0.01 *** |
| 2017 - 2001 | 0.2184 | 0.02775 | 7.872 | <0.01 *** |
| 2018 - 2001 | 0.1571 | 0.02726 | 5.763 | <0.01 *** |
| 2003 - 2002 | -0.1368 | 0.02228 | -6.142 | <0.01 *** |
| 2004 - 2002 | -0.2079 | 0.024 | -8.664 | <0.01 *** |
| 2006 - 2002 | 0.1107 | 0.02738 | 4.043 | <0.01 ** |
| 2008 - 2002 | -0.2421 | 0.02417 | -10.017 | <0.01 *** |
| 2009 - 2002 | 0.1494 | 0.02543 | 5.878 | <0.01 *** |
| 2011 - 2002 | 0.1416 | 0.02802 | 5.053 | <0.01 *** |
| 2012 - 2002 | -0.1656 | 0.02604 | -6.357 | <0.01 *** |
| 2013 - 2002 | -0.1321 | 0.02546 | -5.191 | <0.01 *** |
| 2014 - 2002 | -0.4709 | 0.02824 | -16.676 | <0.01 *** |
| 2015 - 2002 | -0.1241 | 0.02813 | -4.411 | <0.01 ** |
| 2016 - 2002 | -0.3056 | 0.02637 | -11.59 | <0.01 *** |
| 2019 - 2002 | -0.1485 | 0.02466 | -6.024 | <0.01 *** |
| 2005 - 2003 | 0.1965 | 0.02462 | 7.981 | <0.01 *** |
| 2006 - 2003 | 0.2475 | 0.02588 | 9.564 | <0.01 *** |
| 2007 - 2003 | 0.139 | 0.02232 | 6.227 | <0.01 *** |
| 2008 - 2003 | -0.1053 | 0.02246 | -4.687 | <0.01 *** |
| 2009 - 2003 | 0.2863 | 0.02381 | 12.025 | <0.01 *** |
| 2010 - 2003 | 0.1405 | 0.02418 | 5.813 | <0.01 *** |
| 2011 - 2003 | 0.2784 | 0.02656 | 10.482 | <0.01 *** |
| 2014 - 2003 | -0.334 | 0.02679 | -12.469 | <0.01 *** |
| 2016 - 2003 | -0.1688 | 0.02481 | -6.803 | <0.01 *** |
| 2017 - 2003 | 0.1469 | 0.02443 | 6.014 | <0.01 *** |
| 2005 - 2004 | 0.2676 | 0.02619 | 10.218 | <0.01 *** |
| 2006 - 2004 | 0.3186 | 0.02738 | 11.638 | <0.01 *** |
| 2007 - 2004 | 0.2101 | 0.02404 | 8.74 | <0.01 *** |
| 2009 - 2004 | 0.3574 | 0.02543 | 14.056 | <0.01 *** |
| 2010 - 2004 | 0.2116 | 0.02577 | 8.212 | <0.01 *** |
| 2011 - 2004 | 0.3495 | 0.02802 | 12.473 | <0.01 *** |
| 2014 - 2004 | -0.2629 | 0.02824 | -9.311 | <0.01 *** |
| 2016 - 2004 | -0.0977 | 0.02637 | -3.705 | 0.0334 * |
| 2017 - 2004 | 0.218 | 0.02601 | 8.383 | <0.01 *** |
| 2018 - 2004 | 0.1567 | 0.02549 | 6.148 | <0.01 *** |
| 2008 - 2005 | -0.3017 | 0.02634 | -11.454 | <0.01 *** |
| 2012 - 2005 | -0.2252 | 0.02807 | -8.022 | <0.01 *** |
| 2013 - 2005 | -0.1918 | 0.02753 | -6.967 | <0.01 *** |
| 2014 - 2005 | -0.5305 | 0.03012 | -17.614 | <0.01 *** |
| 2015 - 2005 | -0.1837 | 0.03002 | -6.12 | <0.01 *** |
| 2016 - 2005 | -0.3653 | 0.02838 | -12.873 | <0.01 *** |
| 2018 - 2005 | -0.1109 | 0.02756 | -4.024 | 0.0102 * |
| 2019 - 2005 | -0.2082 | 0.02679 | -7.77 | <0.01 *** |
| 2007 - 2006 | -0.1085 | 0.02741 | -3.958 | 0.0143 * |
| 2008 - 2006 | -0.3528 | 0.02753 | -12.817 | <0.01 *** |
| 2010 - 2006 | -0.107 | 0.02894 | -3.696 | 0.0347 * |
| 2012 - 2006 | -0.2762 | 0.02919 | -9.465 | <0.01 *** |
| 2013 - 2006 | -0.2428 | 0.02866 | -8.472 | <0.01 *** |
| 2014 - 2006 | -0.5815 | 0.03116 | -18.665 | <0.01 *** |
| 2015 - 2006 | -0.2347 | 0.03106 | -7.558 | <0.01 *** |
| 2016 - 2006 | -0.4163 | 0.02948 | -14.124 | <0.01 *** |
| 2018 - 2006 | -0.1619 | 0.02869 | -5.644 | <0.01 *** |
| 2019 - 2006 | -0.2592 | 0.02796 | -9.273 | <0.01 *** |
| 2008 - 2007 | -0.2443 | 0.02421 | -10.089 | <0.01 *** |
| 2009 - 2007 | 0.1473 | 0.02547 | 5.783 | <0.01 *** |
| 2011 - 2007 | 0.1394 | 0.02806 | 4.969 | <0.01 *** |
| 2012 - 2007 | -0.1677 | 0.02608 | -6.431 | <0.01 *** |
| 2013 - 2007 | -0.1343 | 0.0255 | -5.268 | <0.01 *** |
| 2014 - 2007 | -0.473 | 0.02827 | -16.732 | <0.01 *** |
| 2015 - 2007 | -0.1262 | 0.02816 | -4.482 | <0.01 ** |
| 2016 - 2007 | -0.3078 | 0.02641 | -11.656 | <0.01 *** |
| 2019 - 2007 | -0.1507 | 0.0247 | -6.102 | <0.01 *** |
| 2009 - 2008 | 0.3916 | 0.02559 | 15.303 | <0.01 *** |
| 2010 - 2008 | 0.2458 | 0.02593 | 9.479 | <0.01 *** |
| 2011 - 2008 | 0.3837 | 0.02817 | 13.622 | <0.01 *** |
| 2013 - 2008 | 0.11 | 0.02562 | 4.293 | <0.01 ** |
| 2014 - 2008 | -0.2288 | 0.02838 | -8.06 | <0.01 *** |
| 2015 - 2008 | 0.118 | 0.02827 | 4.175 | <0.01 ** |
| 2017 - 2008 | 0.2522 | 0.02617 | 9.638 | <0.01 *** |
| 2018 - 2008 | 0.1909 | 0.02565 | 7.441 | <0.01 *** |
| 2019 - 2008 | 0.09356 | 0.02482 | 3.769 | 0.0275 * |
| 2010 - 2009 | -0.1458 | 0.02711 | -5.377 | <0.01 *** |
| 2012 - 2009 | -0.315 | 0.02736 | -11.512 | <0.01 *** |
| 2013 - 2009 | -0.2816 | 0.02681 | -10.505 | <0.01 *** |
| 2014 - 2009 | -0.6203 | 0.02946 | -21.058 | <0.01 *** |
| 2015 - 2009 | -0.2735 | 0.02935 | -9.318 | <0.01 *** |
| 2016 - 2009 | -0.4551 | 0.02768 | -16.444 | <0.01 *** |
| 2017 - 2009 | -0.1394 | 0.02733 | -5.099 | <0.01 *** |
| 2018 - 2009 | -0.2007 | 0.02683 | -7.48 | <0.01 *** |
| 2019 - 2009 | -0.298 | 0.02605 | -11.44 | <0.01 *** |
| 2011 - 2010 | 0.1379 | 0.02955 | 4.666 | <0.01 *** |
| 2012 - 2010 | -0.1693 | 0.02769 | -6.114 | <0.01 *** |
| 2013 - 2010 | -0.1358 | 0.02713 | -5.006 | <0.01 *** |
| 2014 - 2010 | -0.4746 | 0.02976 | -15.947 | <0.01 *** |
| 2015 - 2010 | -0.1278 | 0.02965 | -4.308 | <0.01 ** |
| 2016 - 2010 | -0.3093 | 0.02799 | -11.05 | <0.01 *** |
| 2019 - 2010 | -0.1522 | 0.02639 | -5.77 | <0.01 *** |
| 2012 - 2011 | -0.3072 | 0.02979 | -10.31 | <0.01 *** |
| 2013 - 2011 | -0.2737 | 0.02928 | -9.349 | <0.01 *** |
| 2014 - 2011 | -0.6124 | 0.03173 | -19.305 | <0.01 *** |
| 2015 - 2011 | -0.2656 | 0.03163 | -8.399 | <0.01 *** |
| 2016 - 2011 | -0.4472 | 0.03008 | -14.869 | <0.01 *** |
| 2017 - 2011 | -0.1315 | 0.02976 | -4.418 | <0.01 ** |
| 2018 - 2011 | -0.1928 | 0.0293 | -6.58 | <0.01 *** |
| 2019 - 2011 | -0.2901 | 0.02859 | -10.149 | <0.01 *** |
| 2014 - 2012 | -0.3053 | 0.02999 | -10.179 | <0.01 *** |
| 2016 - 2012 | -0.1401 | 0.02824 | -4.959 | <0.01 *** |
| 2017 - 2012 | 0.1757 | 0.02791 | 6.294 | <0.01 *** |
| 2018 - 2012 | 0.1143 | 0.02742 | 4.169 | <0.01 ** |
| 2014 - 2013 | -0.3387 | 0.02948 | -11.488 | <0.01 *** |
| 2016 - 2013 | -0.1735 | 0.0277 | -6.263 | <0.01 *** |
| 2017 - 2013 | 0.1422 | 0.02736 | 5.199 | <0.01 *** |
| 2015 - 2014 | 0.3468 | 0.03182 | 10.899 | <0.01 *** |
| 2016 - 2014 | 0.1652 | 0.03028 | 5.457 | <0.01 *** |
| 2017 - 2014 | 0.481 | 0.02996 | 16.052 | <0.01 *** |
| 2018 - 2014 | 0.4196 | 0.02951 | 14.219 | <0.01 *** |
| 2019 - 2014 | 0.3223 | 0.0288 | 11.192 | <0.01 *** |
| 2016 - 2015 | -0.1816 | 0.03018 | -6.018 | <0.01 *** |
| 2017 - 2015 | 0.1341 | 0.02986 | 4.492 | <0.01 ** |
| 2017 - 2016 | 0.3157 | 0.02821 | 11.191 | <0.01 *** |
| 2018 - 2016 | 0.2544 | 0.02773 | 9.173 | <0.01 *** |
| 2019 - 2016 | 0.1571 | 0.02697 | 5.825 | <0.01 *** |
| 2019 - 2017 | -0.1586 | 0.02662 | -5.96 | <0.01 *** |
| 2019 - 2018 | -9.73E-02 | 0.02611 | -3.727 | 0.0309* |
| **Wasting - only significant rows shown** | | | | |
| 2001 - 1999 | 0.736269 | 0.203982 | 3.609 | 0.0460 * |
| 2007 - 2001 | -0.76881 | 0.195411 | -3.934 | 0.0145 * |
| 2015 - 2007 | 0.740979 | 0.206086 | 3.595 | 0.0482 * |
| **Epiphytes - only significant rows shown** | | | | |
| 1999 - 1998 | -1.09483 | 0.28367 | -3.86 | 0.0181 * |
| 2012 - 1998 | 0.76845 | 0.16436 | 4.676 | <0.01 *** |
| 2000 - 1999 | 1.14611 | 0.27725 | 4.134 | <0.01 ** |
| 2003 - 1999 | 1.50196 | 0.25781 | 5.826 | <0.01 *** |
| 2004 - 1999 | 1.60285 | 0.2602 | 6.16 | <0.01 *** |
| 2005 - 1999 | 1.32213 | 0.27394 | 4.826 | <0.01 *** |
| 2006 - 1999 | 1.4345 | 0.2745 | 5.226 | <0.01 *** |
| 2009 - 1999 | 1.14848 | 0.27831 | 4.127 | <0.01 ** |
| 2010 - 1999 | 1.0815 | 0.28262 | 3.827 | 0.0202 * |
| 2011 - 1999 | 1.53701 | 0.27282 | 5.634 | <0.01 *** |
| 2012 - 1999 | 1.86329 | 0.25881 | 7.199 | <0.01 ** |
| 2013 - 1999 | 1.06991 | 0.2816 | 3.799 | 0.0215 * |
| 2015 - 1999 | 1.54793 | 0.27211 | 5.689 | <0.01 ** |
| 2017 - 1999 | 1.6393 | 0.26396 | 6.21 | <0.01 *** |
| 2018 - 1999 | 1.2641 | 0.27417 | 4.611 | <0.01 *** |
| 2019 - 1999 | 1.3574 | 0.2683 | 5.059 | <0.01 *** |
| 2012 - 2000 | 0.71718 | 0.153 | 4.687 | <0.01 *** |
| 2003 - 2001 | 0.85926 | 0.19915 | 4.315 | <0.01 ** |
| 2004 - 2001 | 0.96014 | 0.20223 | 4.748 | <0.01 *** |
| 2006 - 2001 | 0.79179 | 0.22033 | 3.594 | 0.0446 * |
| 2011 - 2001 | 0.8943 | 0.21823 | 4.098 | <0.01 ** |
| 2012 - 2001 | 1.22058 | 0.20045 | 6.089 | <0.01 *** |
| 2015 - 2001 | 0.90522 | 0.21735 | 4.165 | <0.01 ** |
| 2017 - 2001 | 0.9966 | 0.20705 | 4.813 | <0.01 *** |
| 2003 - 2002 | 0.87078 | 0.17836 | 4.882 | <0.01 *** |
| 2004 - 2002 | 0.97167 | 0.18179 | 5.345 | <0.01 *** |
| 2006 - 2002 | 0.80332 | 0.20173 | 3.982 | 0.0107 * |
| 2011 - 2002 | 0.90583 | 0.19944 | 4.542 | <0.01 ** |
| 2012 - 2002 | 1.23211 | 0.17981 | 6.852 | <0.01 ** |
| 2015 - 2002 | 0.91675 | 0.19848 | 4.619 | <0.01 *** |
| 2017 - 2002 | 1.00813 | 0.18714 | 5.387 | <0.01 *** |
| 2019 - 2002 | 0.72623 | 0.19321 | 3.759 | 0.0255 * |
| 2007 - 2003 | -0.93075 | 0.18332 | -5.077 | <0.01 *** |
| 2008 - 2003 | -0.82561 | 0.17669 | -4.673 | <0.01 *** |
| 2016 - 2003 | -0.76599 | 0.19498 | -3.928 | 0.0138 * |
| 2007 - 2004 | -1.03164 | 0.18666 | -5.527 | <0.01 *** |
| 2008 - 2004 | -0.92649 | 0.18016 | -5.143 | <0.01 *** |
| 2014 - 2004 | -0.83456 | 0.21155 | -3.945 | 0.0127 * |
| 2016 - 2004 | -0.86688 | 0.19813 | -4.375 | <0.01 ** |
| 2007 - 2005 | -0.75092 | 0.20539 | -3.656 | 0.0376 * |
| 2012 - 2005 | 0.54116 | 0.14693 | 3.683 | 0.0325 * |
| 2007 - 2006 | -0.86329 | 0.20613 | -4.188 | <0.01 ** |
| 2008 - 2006 | -0.75814 | 0.20026 | -3.786 | 0.0232 * |
| 2011 - 2007 | 0.9658 | 0.20389 | 4.737 | <0.01 *** |
| 2012 - 2007 | 1.29208 | 0.18473 | 6.994 | <0.01 *** |
| 2015 - 2007 | 0.97672 | 0.20295 | 4.813 | <0.01 ** |
| 2017 - 2007 | 1.0681 | 0.19187 | 5.567 | <0.01 *** |
| 2019 - 2007 | 0.7862 | 0.1978 | 3.975 | 0.0119 * |
| 2011 - 2008 | 0.86065 | 0.19795 | 4.348 | <0.01 ** |
| 2012 - 2008 | 1.18693 | 0.17816 | 6.662 | <0.01 *** |
| 2015 - 2008 | 0.87157 | 0.19698 | 4.425 | <0.01 ** |
| 2017 - 2008 | 0.96295 | 0.18555 | 5.19 | <0.01 *** |
| 2012 - 2009 | 0.71481 | 0.15492 | 4.614 | <0.01 *** |
| 2012 - 2010 | 0.78178 | 0.16254 | 4.81 | <0.01 *** |
| 2016 - 2011 | -0.80104 | 0.21444 | -3.736 | 0.0279 * |
| 2013 - 2012 | -0.79337 | 0.16076 | -4.935 | <0.01 *** |
| 2014 - 2012 | -1.095 | 0.20985 | -5.218 | <0.01 *** |
| 2016 - 2012 | -1.12732 | 0.19631 | -5.742 | <0.01 *** |
| 2018 - 2012 | -0.59919 | 0.14736 | -4.066 | <0.01 ** |
| 2019 - 2012 | -0.50588 | 0.13612 | -3.716 | 0.0294 * |
| 2017 - 2014 | 0.87101 | 0.21616 | 4.029 | <0.01 ** |
| 2016 - 2015 | -0.81196 | 0.21354 | -3.802 | 0.0216 * |
| 2017 - 2016 | 0.90333 | 0.20305 | 4.449 | <0.01 ** |
